# Electrophilic Compounds in the Human Diet and Their Role in the Induction of the Transcription Factor NRF2

**DOI:** 10.3390/ijms25063521

**Published:** 2024-03-20

**Authors:** Celia María Curieses Andrés, José Manuel Pérez de la Lastra, Elena Bustamante Munguira, Celia Andrés Juan, Francisco J. Plou, Eduardo Pérez Lebeña

**Affiliations:** 1 Hospital Clínico Universitario of Valladolid, Avda. de Ramón y Cajal, 3, 47003 Valladolid, Spain; cmcurieses@gmail.com (C.M.C.A.); ebustamante@saludcastillayleon.es (E.B.M.); 2 Institute of Natural Products and Agrobiology, CSIC-Spanish Research Council, Avda. Astrofísico Fco. Sánchez, 3, 38206 La Laguna, Spain; jm.perezdelalastra@csic.es; 3 Cinquima Institute and Department of Organic Chemistry, Faculty of Sciences, Valladolid University, Paseo de Belén, 7, 47011 Valladolid, Spain; 4 Institute of Catalysis and Petrochemistry, CSIC-Spanish Research Council, 28049 Madrid, Spain; fplou@icp.csic.es; 5 Sistemas de Biotecnología y Recursos Naturales, 47625 Valladolid, Spain; info@glize.eu

**Keywords:** electrophilic compounds, polyphenols, hydrogen sulfide, NRF2/ARE axis, Keap1, organic sulfur, Michael acceptors

## Abstract

The phrase “Let food be thy medicine…” means that food can be a form of medicine and medicine can be a form of food; in other words, that the diet we eat can have a significant impact on our health and well-being. Today, this phrase is gaining prominence as more and more scientific evidence suggests that one’s diet can help prevent and treat disease. A diet rich in fruits, vegetables, whole grains, and lean protein can help reduce the risk of heart disease, cancer, diabetes, and other health problems and, on the other hand, a diet rich in processed foods, added sugars, and saturated fats can increase the risk of the same diseases. Electrophilic compounds in the diet can have a significant impact on our health, and they are molecules that covalently modify cysteine residues present in the thiol-rich Keap1 protein. These compounds bind to Keap1 and activate NRF2, which promotes its translocation to the nucleus and its binding to DNA in the ARE region, triggering the antioxidant response and protecting against oxidative stress. These compounds include polyphenols and flavonoids that are nucleophilic but are converted to electrophilic quinones by metabolic enzymes such as polyphenol oxidases (PPOs) and sulfur compounds present in foods such as the Brassica genus (broccoli, cauliflower, cabbage, Brussel sprouts, etc.) and garlic. This review summarizes our current knowledge on this subject.

## 1. Introduction

The quote “Let food be thy medicine and medicine be thy food”, a translation of the Latin phrase “Cibus sit medicina tua, et medicina sit cibus tuus”, is erroneously attributed to Hippocrates, the founder of medicine, even though it does not appear in any of his written works [1]. Despite its ancient origins, this phrase emphasizes the need for good nutrition and the different medicinal properties of the various nutrients. A healthy lifestyle and a balanced diet are essential for optimal health and the prevention of disease [2].

An essential aspect of a balanced diet is ensuring adequate food intake in order to meet one’s nutritional needs. Eating certain diets can exacerbate many diseases such as cancer, diabetes, cardiovascular disease, and high blood pressure. Conversely, some diets have been shown to reduce the likelihood of developing certain diseases [3]. Switching to a plant-based diet has been shown to have several benefits, such as reducing the risk of cancer, helping to cure certain diseases, and protecting against certain diseases [4].

The most important components of a plant-based diet are vegetables, legumes, whole grains, fruits, nuts, and seeds. A moderate number of low-fat dairy products can be included in the diet, while fish and poultry can make up to twenty percent of the total food intake, depending on personal preference. These meals are nutrient-dense and contain a wide range of beneficial compounds such as vitamins, polyphenols, minerals, amino acids, proteins, healthy fats, fiber, and phytochemicals. These substances are essential for the general well-being of humans and animals [5].

The statement “Let food be your medicine…” provides a solid foundation for discussing the lifestyle changes needed to prevent and treat disease and emphasizes the important role of good nutrition in disease prevention without suggesting that standard treatment is unnecessary. Therefore, it is beneficial to heed this advice to maximize the benefits of a nutritious diet [6].

Electrophilic compounds (ECs) are molecules that may have beneficial health effects due to their ability to interact with various proteins and cellular components [7]. Some areas where ECs have a positive impact are as follows:

- Some ECs have antioxidant properties, which help neutralize free radicals and protect against oxidative damage to cells [8];
- ECs can modulate the inflammatory response by interacting with proteins involved in inflammation. This may help to reduce chronic inflammation and improve health [9];
- Some ECs have been shown to have anti-tumor effects by targeting specific proteins in cancer cells. These compounds may help prevent the growth and spread of cancer [10];
- ECs can induce the expression of phase II enzymes. These enzymes help detoxify the body by removing harmful substances [11];
- ECs found in olive oil and garlic exert protective effects on the cardiovascular system by improving endothelial function and reducing inflammation [12].

In the following sections, we will explore the extent of this concept and the current scientific evidence underpinning this popular notion.

## 2. NRF2 and Keap1

NRF2 was discovered in 1994 by P. Moi et al. [13] and is a transcription factor called Nuclear Factor Erythroid 2-Related Factor 2, which regulates the activation of genes that protect cells from oxidative damage, inflammation, and toxicity. NRF2 is a transcription factor with a cap-“n”-collar (CNC) motif and a basic leucine zipper structure (bZip). It consists of seven NRF2 ECH homology (Neh) domains that are highly conserved [14].

**Figure 1:**
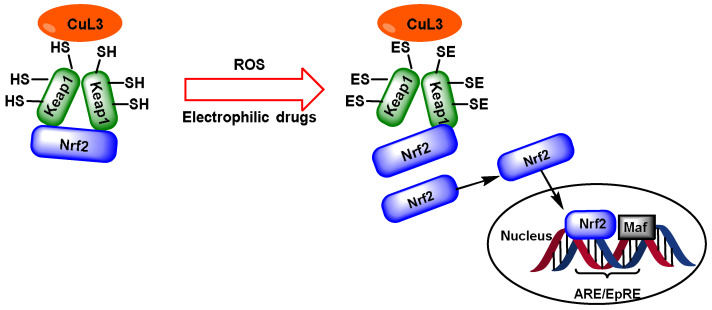
When cells are exposed to ROS and electrophiles, the thiol groups of cysteine residues in Keap1 are directly modified, leading to a decrease in Keap1-dependent NRF2 ubiquitination and a rapid accumulation of newly synthesized NRF2. Subsequently, NRF2 is stabilized, translocates to the nucleus and forms a heterodimer with small Maf proteins, and activates target genes for cytoprotection through antioxidant response elements (AREs) or electrophile responsive elements (EpREs).

NRF2 can be activated by various stimuli, including free radicals, polyphenols, toxic substances, infections, exercise, and diet [15]. When NRF2 is activated, it attaches to a specific DNA region called the antioxidant response element (ARE) [16] (Figure 1).

This process leads to the activation of genes responsible for producing antioxidant and detoxifying enzymes and other protective proteins that protect cells from injury. The NRF2 hypothesis has never been proven in humans due to the slow progression of the monitoring of the NRF2 activity in vivo [17]. In vitro studies have shown that NRF2 could be beneficial in the prevention of various diseases, such as cancer, cardiovascular disease, diabetes, Alzheimer’s disease, and Parkinson’s disease [18,19,20]. In addition, there is a direct link between NRF2 and longevity. Accordingly, animals with elevated NRF2 levels tend to live longer than those with low NRF2 levels [21].

**Figure 2:**
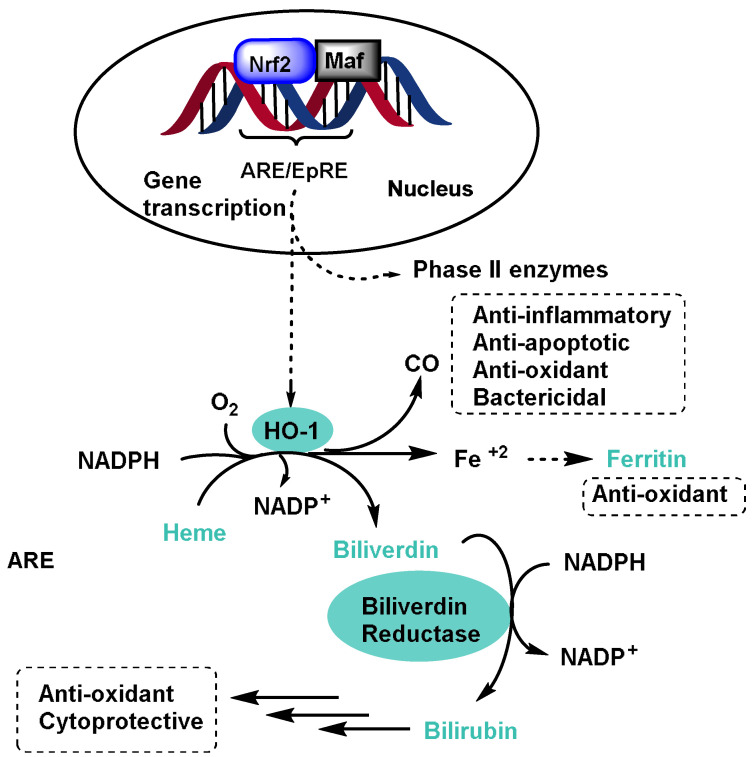
HO-1 enzyme activity. HO-1 enzyme activity generates carbon monoxide (CO), free iron (Fe^2+^), and biliverdin IXa in equimolar amounts. Biliverdin is rapidly metabolized to bilirubin by biliverdin reductase. Free iron, which can stimulate free radical formation, is rapidly scavenged by ferritin (an antioxidant). At physiological concentrations, CO has cytoprotective, anti-inflammatory, antioxidant, and bactericidal properties [26,27].

NRF2 induces the expression of Heme Oxygenase-1 (HO-1) [22], which leads to an increase in phase II enzymes [23] and also inhibits the NLRP3 inflammasome component [24], which is mainly found in macrophages and recognizes damaged cell products in order to trigger an immune response [25] (Figure 2).

**Figure 3:**
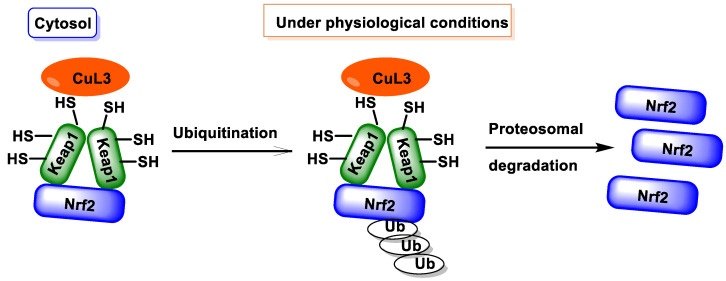
Under normal conditions, NRF2 is ubiquitinated by Keap1 and its degradation is carried out by proteasomes.

Under basal conditions (no stress), NRF2 is maintained in the cytoplasm by proteins that rapidly degrade it. Under oxidative stress, NRF2 travels to the nucleus, where it binds to a DNA promoter and initiates the transcription of antioxidant and protective genes and their proteins [28] (Figure 3).

Keap1 is a substrate adaptor protein that helps in this process. Keap1 is responsible for sequestering NRF2 in the cytoplasm, while Cullin 3 is responsible for labeling NRF2 for degradation by ubiquitination [29]. After NRF2 is ubiquitinated, it is transported to the proteasome for destruction and its components are subsequently recycled. Under typical conditions, the half-life of NRF2 is only twenty minutes [30].

NRF2 regulates about two thousand genes related to the redox balance and many metabolic pathways that may not be directly related to oxidative stress [31]. Over the past 25 years, the number of factors affecting NRF2 activation, nuclear translocation, and inactivation has increased significantly [32]. NRF2 triggering induces the transcription of genes encoding cytoprotective proteins and enzymes [33], such as sulfiredoxin 1 (SRXN1) and thioredoxin reductase 1 (TXNRD1), that support the reduction and recovery of peroxiredoxins, which are proteins important in the detoxification of highly reactive peroxides such as hydrogen peroxide and peroxynitrite [34], and multidrug-resistance-associated proteins (Mrps), which are important membrane transporters that expel certain compounds from various organs into bile or plasma with subsequent excretion in feces or urine, respectively [35]. NRF2 has been shown to positively regulate Mrps and alterations in its expression can drastically alter the pharmacokinetics and toxicity of compounds [36].

The Keap1 protein plays a key role in controlling NRF2 deactivation. This protein acts as a substrate adaptor for a Cul3-containing E3 ubiquitin ligase [37]. NRF2 is ubiquitinated and destroyed by the Keap1–Cul3–E3 ligase complex when activated, leading to the ubiquitination and degradation of NRF2 [38]. The function of this complex is inhibited when it is exposed to oxidative stress or foreign substances with electrophilic properties. As a result, the NRF2 protein accumulates and migrates into the nucleus, leading the activation of its target genes [15].

Keap1 contains four unique protein domains: the N-terminal Broad complex, the Tramtrack and Bric-à-Brac (BTB) domain, the intermediate region (IVR) domain, the double glycine repeat (DGR) domain, and the C-terminal domain (CTR) [39]. The BTB domain contains the Cys151 residue, a specific cysteine that is critical for stress sensing. The IVR domain contains two crucial cysteine residues, Cys273 and Cys288, which are necessary for stress sensing in the second group of cysteines [40]. Keap1 interacts with NRF2 in the β-propeller structure formed by the cooperation of the DGR and CTR domains [41].

In 1999, Itoh et al. [42] discovered the Keap1 protein and its ability to inhibit the function of NRF2. Eggler et al. [43] also reported similar findings in 2005, providing the most widely accepted model for the translocation of NRF2 to the nucleus. This concept explains how the cysteines in Keap1 are subjected to an electrophilic attack, reducing their binding affinity for NRF2. Eggler and colleagues have shown that the binding of NRF2 to the Keap1 protein remains intact, but Keap1 stops ubiquitinating NRF2 and starts to ubiquitinate the complex, leading to degradation at the proteasome. Turpaev [44] found that the newly produced NRF2 can no longer find Keap1 to bind to due to the decrease in the Keap1 concentration. Consequently, it can easily enter the cell nucleus and attach to the ARE region.

The interaction between the Keap1 and NRF2 proteins is complex and should be carefully considered in order to avoid oversimplification of the model. The Neh2 domain of NRF2 binds with two Keap1 molecules in a 2:1 stoichiometry, resulting in the formation of a very stable complex [41]. Electrophiles cause significant conformational changes in Keap1, but NRF2 remains trapped due to the blocking of the complex formed. Simply put, the NRF2 component cannot escape. Since the ubiquitination machinery cannot dissociate, NRF2 remains bound and is not released [45]. The NRF2 that can enter the nucleus is only that which is produced de novo, as it cannot find a Keap1 protein to which it can bind. An increase in NRF2 levels can only occur if Keap1 has previously been degraded [46].

For the NRF2 to bind to the ARE region, it must undergo acetylation and phosphorylation while avoiding glycation [47]. The CREB-binding protein, or CBP, is a transcriptional coactivator for CREB. Acetylation of NRF2 is responsible for increasing its stability, promoting nuclear translocation, and enhancing its activity [48]. The histone deacetylases SIRT1 and SIRT2 are expected to decrease the transcriptional activity of NRF2 [49]. Phosphorylation of NRF2 by MAPK kinases at serine 40 contributes to its migration into the nucleus. Phosphorylation by CK2 in the Neh4 and Neh5 domains is critical for ARE binding and transcriptional activity. Both processes are crucial to the protein’s ability to perform its tasks effectively [50,51].

A link between the activation of NRF2 transcription and the production of HO-1 and NADP(H)-quinone oxidoreductase 1 (NQO1) has been demonstrated [52]. However, many NRF2 target genes are also influenced by other transcription factors. The bZip protein BACH1 represses the HMOX1 gene, which encodes HO-1. This property is the main element that inhibits NRF2 activation of HMOX1 transcription [53]. To effectively activate HMOX1, BACH1 must first be deactivated. Several electrophilic chemicals traditionally thought to stimulate NRF2 inhibit BACH1 in practice [54,55].

NRF2 and BACH1 are two transcription factors that compete for binding to the ARE [56]. Binding of BACH1 to the ARE inhibits transcription, while binding of NRF2 to the ARE stimulates the transcription of these genes [57]. The competition between NRF2 and BACH1 for the ARE plays a crucial role in maintaining the redox balance and antioxidant response in cells. The link between NRF2 and BACH1 plays a crucial role in the pathogenesis of diseases such as cancer [58] and chronic obstructive pulmonary disease (COPD) [59].

NF-κB is a transcription factor involved in the regulation of cellular immune responses triggered by infection and excessive oxidative stress leading to increased inflammation [60]. NRF2 expression can inhibit NF-κB activation. It is known that NF-κB can inhibit NRF2. Overexpression of NF-κB may be the critical point at which increased oxidative stress leads to inflammation [61].

Phase II enzymes play a crucial role in cellular biotransformation, which involves detoxifying the following xeno- and endobiotic compounds:

- The glutathione S-transferase (GST) family, which includes cytosolic, mitochondrial, and microsomal enzymes that catalyze the conjugation of GSH to endogenous electrophiles and xenobiotics [62]. After detoxification by the GST-catalyzed glutathione (GSH) conjugation, the body can eliminate potentially harmful and toxic compounds. GSTs are induced by NRF2 activation and represent an important detoxification pathway [63];
- The UDP-glucuronosyltransferase (UGT) family, which catalyzes the conjugation of a glucuronic acid moiety to a variety of endogenous and exogenous substances, making them more water soluble and easily excreted. Important substrates for glucuronidation include bilirubin and acetaminophen [64]. NRF2 has been shown to induce UGT1A1 and UGT1A6 [65];
- N-acetyltransferase (NAT), acetylate aromatic amines, and hydrazines participate in the metabolism of drugs, carcinogens, and arylamines. Genetic variations in NATs can affect drug efficacy and toxicity [66];
- Sulfotransferases (SULTs) transfer sulfate groups to substrates, enhancing their water solubility. These enzymes are crucial for detoxifying phenolic compounds, drugs, and hormones [67];
- Epoxide hydrolase (EH), although traditionally considered a phase I enzyme, also plays a role during phase II. It detoxifies epoxides formed during phase I reactions, preventing their harmful effects [68];
- Heme oxygenase-1 (HO-1) degrades heme into biliverdin, carbon monoxide, and iron. It has antioxidant and anti-inflammatory properties, protecting cells from oxidative stress. HO-1 protects against a variety of pathologies, including sepsis, hypertension, atherosclerosis, acute lung injury, kidney injury, and pain [69];
- NQO1 reduces quinones, preventing their conversion into reactive oxygen species. It contributes to the cellular defense against oxidative damage [70];
- Glutamate cysteine ligase (GCL), although not directly involved in biotransformation, is critical for GSH biosynthesis, a potent antioxidant that protects cells from oxidative stress [71];
- Ferritin is not an enzyme but is the main intracellular iron storage protein. It helps regulate iron levels and prevents iron-induced oxidative damage [72].

These phase II enzymes play major roles in detoxification, cytoprotection, and cancer prevention. Their coordinated induction through the Keap1–NRF2–ARE signaling pathway ensures cellular resilience against harmful agents [73].

## 3. Role of NRF2 in Senescence

Cellular senescence is a state of irreversible cessation of cell division that is associated with aging and age-related diseases [74]. It is defined by the permanent cessation of cell division, which leads to an accumulation of senescent cells in tissues and organs [75]. The shape of dormant cells changes, leading to an increase in the production of reactive oxygen species (ROS) [76] and the release of pro-inflammatory factors [77]. These morphological changes are just some of the variations that these cells exhibit. Cellular senescence can be induced by various methods, such as the following:

- Telomeres are protective structures located at the ends of chromosomes. Their length decreases with each successive cell cycle. Senescence happens when the length of the telomeres becomes critically short [78];
- Significant DNA damage that stops the replication of damaged cellular material [79];
- Oxidative stress and restricted nutrition can contribute to cell senescence [80].

NRF2 regulates both the inflammatory response and cellular senescence [81]. Atherosclerosis, Alzheimer’s disease, and cancer are among the age-related diseases associated with chronic inflammation, a common feature of aging associated with this condition [82].

Autophagy is an essential process that occurs in eukaryotic cells and degrades damaged or unneeded cellular material [83]. Organelles that generate excessive ROS in aging cells are responsible for the cellular remodeling triggered by this phenomenon. This process is crucial for the adaptation to environmental conditions and the restructuring of cells [84].

In the absence of autophagy, Keap1 is deleted, leading to the activation of NRF2 and an increase in the expression of the ARE gene. The connections between NRF2, ARE, and Keap1 are crucial for the regulation of autophagy. Autophagy is impaired in disease by the interaction of mutant proteins and an increase in ROS [84].

Mitosis is a fundamental process of life that involves cell division and leads to the formation of two daughter cells that are genetically identical to the parent cell. It is essential for the growth, development, and repair of cells. The regulation of mitochondrial quality, which is influenced by mitochondrial dynamics and biogenesis, is directly linked to cell mitosis [85].

Recent studies have shown that PTEN-induced kinase 1 (PINK1) has a critical function in maintaining mitochondrial integrity and protecting cells from oxidative stress. Research shows that NRF2 positively influences PINK1 production and that the NRF2–PINK1 signaling pathway is critical for cell survival [86].

Recent studies have revealed that PINK1 is an essential factor controlling mitochondrial quality and protecting cells from oxidative stress. The results indicate that PINK1 expression is positively regulated by NRF2 and that the NRF2–PINK1 signaling axis is deeply involved in cell survival [87].

Parkin acts as a ubiquitin ligase and directs other proteins for degradation by the proteasome, a cellular mechanism responsible for recycling damaged or dysfunctional proteins [88].

Parkin is a protein that specifically identifies damaged mitochondria, the essential components of the cell, and directs them for removal. Parkin and NRF2 are proteins that have been studied in the context of Parkinson’s disease [89]. Parkin and NRF2 work together in many ways to accomplish their tasks. Parkin can facilitate the degradation of the protein in order to control the activity of NRF2. NRF2 may enhance the function of Parkin by increasing the expression of genes related to mitochondrial biogenesis and quality control. Parkin and NRF2 have the potential to be therapeutic targets for Parkinson’s disease [90].

For stem cells, renewal and differentiation are critical for maintaining balance and repairing tissues as the body ages [91]. It is widely known that, as we age, the effectiveness of stem cells in many organs and tissues declines [92]. NRF2 appears to be involved in the rejuvenation of stem cells as part of the aging process [93]. Several studies have shown that enhancing NRF2 could improve neural progenitor cell (NSPC) survival and function through positive regulation [94].

## 4. Electrophilic Compounds Can Interfere with the Keap1 Protein

**Figure 4:**
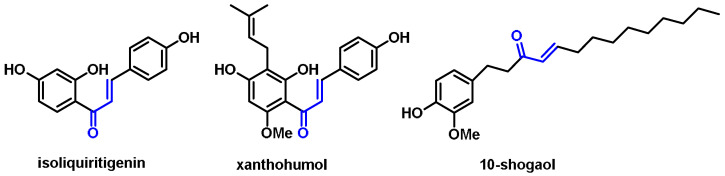
Molecular structure of xanthohumol, isoliquiritigenin, and 10-shogaol.

The thiol groups of the Keap1 sensor can interact with several phytochemicals, each of which can cause NRF2 activation [95]. Keap1 has at least 25 reactive Cys-SH thiols, leading to a variety of effects on the sensor’s response to electrophilic substances [96]. Research shows that the amino acid residue C151 in human Keap1 is the most reactive of all residues when it comes to Michael acceptors. Xanthohumol, isoliquiritigenin, and 10-shogaol (Figure 4), which all act as acceptors in the Michael addition process, showed different degrees of binding to Keap1 in their individual responses. Luo et al. (2007) [97] found that the alkylation of C151 was not affected by any of the three electrophiles. Human Keap1 has the major reactive thiols at positions C151, C266, xanthohumol, C319, and C615. The catalysts C151, C257, and C368 enabled the synthesis of 10-shogaol [97]. The protein C151 is necessary for the production of sulforaphane [98] and falcarindiol [99].

### 4.1. Electrophilic Dietary Compounds that Are Not Michael Acceptors

Organosulfides are a specific group of phytochemicals that are highly effective at activating NRF2. This group includes the following phytochemicals:

- Isothiocyanates (ITCs) are produced by many plants belonging to the *Brassicaceae*, *Capparaceae*, and *Caricaceae* families. Sulforaphane, sinigrin, allyl isothiocyanate, and methyl isothiocyanate are all types of ITCs [100];
- The compounds obtained from garlic. Allium sativum, a culinary plant, is known for its strong odor and unique taste. The main compounds extracted from garlic are allicin, which is formed when allicin is degraded by crushing or chopping enzymes, allyl sulfides, which are formed from the decomposition of allicin, ajoene, a more stable derivative of allicin formed by chemical reactions, and s-allylcysteine (SAC), which is formed from allicin [101].

**Figure 5:**
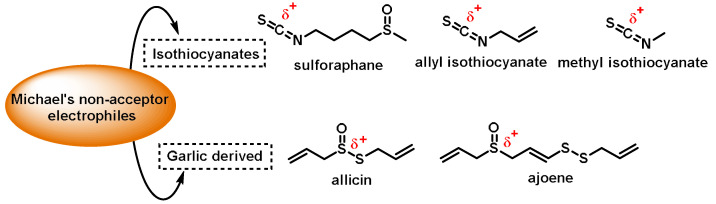
Molecular structure of electrophiles that are not Michael acceptors.

Although these sulfur compounds exhibit electrophilic behavior, they do not have Michael acceptor properties (Figure 5).

### 4.2. Electrophilic Dietary Compounds That Are Michael Acceptors

**Figure 6:**
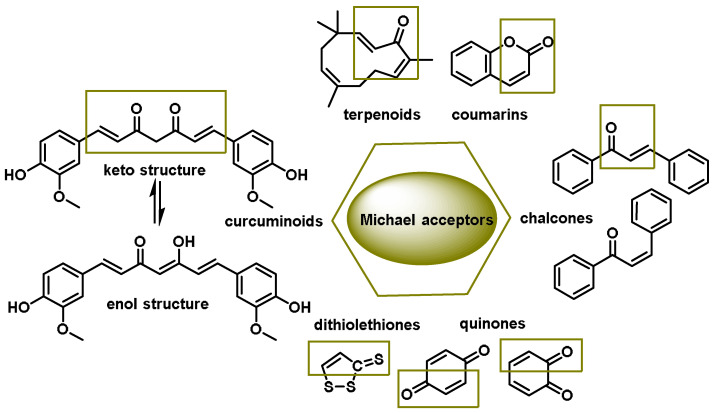
Molecular structures of Michael acceptor compounds.

Dithiolethiones, curcuminoids, chalcones, quinones, terpenoids, and coumarins are all types of Michael acceptors. Michael acceptors are compounds with double bonds that are near electron-withdrawing groups. The preferred electronic groups include carbonyl, nitrile, and nitro groups [102]. A molecule with an α,β-unsaturated carbonyl group is referred to as a Michael acceptor [103] (Figure 6).

**Figure 7:**
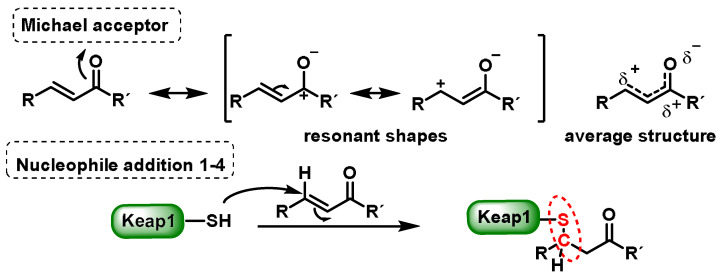
Michael acceptor reaction with the thiol group of cysteines in the Keap1 protein.

Michael acceptors can undergo an addition reaction with the thiol group of cysteines. The sulfur of the cysteine residue reacts with an activated carbon in the Michael acceptor molecule [104]. The sulfur of the cysteine molecule reacts in a nucleophilic 1–4 addition with the carbon of the Michael acceptor chemical. This reaction, which is part of a broader category of conjugate additions, can efficiently form S–C bonds under mild conditions (Figure 7).

Michael acceptor molecules can bind with nucleophilic residues in proteins, forming covalent complexes and impeding the protein’s properties in order to play a physiological role. So, they can regulate the Keap1–NRF2–ARE and NF-κB pathways and can be used in diseases such as inflammation, cancer, and oxidative stress [105]. Michael acceptor molecules can change the biological conformation of proteins or enzymes containing nucleophilic groups, thus inducing consequent physiological variations, and the following compounds from this group have now been approved as drugs for a variety of treatments [106]:

- Dithiolethiones are sulfur-containing pentacyclic compounds that exhibit anti-inflammatory, antithrombotic, antioxidant, and chemotherapeutic properties. Researchers are investigating their potential as cancer therapies to prevent cancer in humans, both in the laboratory and in clinical settings [107];
- Curcumin is the major compound in the curcuminoids extracted from turmeric and is a natural polyphenolic molecule. Curcumin contains two α,β-unsaturated residues attached to a carbonyl, so it is a Michael acceptor. Curcumin exists in two tautomeric forms (Keto and Enol). Keto is a solid, but Enol is a liquid [108];
- Chalcones are aromatic ketones that serve as building blocks for several important biological compounds known as bioactive substances. They are present in a variety of foods, such as vegetables, fruits, and teas, as well as in fluorescent materials and chemical intermediates [109]. Chalcones can occur in two forms (cis and trans isomers). Chalcones have a simple chemical compound structure. The trans isomer is thermodynamically more stable. The presence of the Michael acceptor, an α,β-unsaturated carbonyl system, is a key factor in the observed biological activity. They can easily obtain the Michael adduct due to the facile formation of covalent bonds with nucleophiles such as the sulfhydryl group of cysteine residues [110]. Chalcones are predominantly soft electrophiles and soft nucleophiles that have an affinity for thiol moieties. Chalcones are used in medicinal chemistry for many purposes, e.g., as antioxidants, anticancer drugs, antidiabetics, antiviral agents, and antimalarials [111];
- Quinones are a group of compounds present in various bacteria, fungi, and plants. Quinones are ECs that act as Michael acceptors and are stabilized by conjugation. They also act as oxidizing agents and their effect can sometimes be reversed [112];
- Coumarins are phenolic compounds derived from cinnamic acid and are present in several plant species, including edible, medicinal, and aromatic plants from different botanical families, as well as in fungi and bacteria. Coumarins belong to the class of benzopyrans and are present in a variety of medicinal plants [113,114,115,116]. They exhibit a broad spectrum of pharmacological effects, such as anti-inflammatory, anticoagulant, anticancer, antibacterial, antimalarial, antifungal, antiviral, ulcerogenic, and antihypertensive effects. They are present in various parts of plants, such as roots, seeds, nuts, flowers, and fruits, either as heterosides or in their free form. Coumarins are classified as Michael acceptors as they contain an α,β-unsaturated carbonyl [117];
- Terpenoids are the most abundant category of phytochemicals. They can be present in different plant species and play various biological and metabolic roles in living organisms. Green plants, especially those with flowers, have a significantly large number of terpenoid compounds compared with other living organisms [118]. Terpenoids ingested with food have a greater influence on the modulation of the Keap1–NRF2–ARE signaling pathway [119]. Zerumbone is a monocyclic sesquiterpene compound extracted from the rhizomes of Zingiber zerumbet Smith. The compound has three double bonds, two of which are conjugated and one that is isolated, as well as a conjugated carbonyl group. It is structured in an 11-membered ring configuration [120].

Several foods exhibit physiological activity because they contain electrophilic groups in their structure or their derivatives. These compounds can bind to nucleophilic residues in proteins, resulting in a therapeutic benefit with low toxicity in many diseases [121].

## 5. Role of Hydrogen Sulfide

Hydrogen sulfide (H_2_S) is recognized as a physiological modulator in mammals and acts as an endogenous gasotransmitter that can pass through cell membranes without the need for a specific transporter [122]. H_2_S was previously thought to be a toxic waste product of biological metabolism. Recent studies have shown that H_2_S acts as a signaling molecule in various physiological processes, including regulation of the immune system, neuroprotection, and cardiovascular health [123]. Various enzymes with known molecular regulatory mechanisms can generate H_2_S from L-cysteine, D-cysteine, homocysteine, cystathione, and 3-mercaptopyruvate [124]. In mammalian cells, H_2_S is produced through the following two main mechanisms:

- Enzymatic synthesis in tissues is carried out by certain enzymes, such as cystathionine-γ-lyase (CSE) and 3-mercaptopyruvate sulfurtransferase (3MST), that convert cysteine or other sulfur-containing molecules into H_2_S. Organs and tissues such as the brain, heart, kidneys, and lungs contain these enzymes [125];
- Bacteria in the gut play an important role in the production of much of the H_2_S in the body through their metabolism in the digestive system. These bacteria, such as *Escherichia coli* and *Bacteroides*, use sulfur-containing amino acids to produce H_2_S, which then enters the bloodstream and is distributed throughout the body [126].

Enzymatic synthesis in the tissues is mainly responsible for regulating local H_2_S signaling, while bacterial metabolism in the gut is mainly involved in the production of systemic H_2_S [127].

In 1996, it was shown that cystathionine-β-synthase (CBS) in the brain can produce H_2_S. This process facilitates the initiation of long-term potentiation in the hippocampus by increasing the stimulation of NMDA receptors [128]. In 1997, the enzyme cystathionine gamma-lyase (CSE) was shown to be present in the thoracic aorta, portal vein, and ileum and to produce H_2_S [129]. H_2_S has also been shown to have the ability to relax these tissues. 3-mercaptopyruvate sulfurtransferase, or 3-MST, is an enzyme responsible for the formation of hydrogen sulfide. It is present in neurons, in the vascular endothelium, and in the mitochondria of almost all cells worldwide. The H_2_S produced by 3-MST in the mitochondria not only replenishes GSH levels but also reduces the production of ROS in these organelles [130].

CSE promotes the production of H_2_S, particularly in the pancreas, adipose tissue, liver, cardiovascular system, and respiratory system. CBS is the main factor responsible for the formation of H_2_S in the central nervous system [131].

The gut microbiota produces H_2_S, which significantly affects the physiological processes of the host organism and the microbial community [132]. Intestinal cells produce sulfide using CBS and CSE enzymes, while some intestinal bacteria use sulfate in their respiratory chain to produce H_2_S. This indicates the presence of an additional source of sulfide in this tissue. Excess gas poses a threat to organs in the digestive tract [126]. The production of H_2_S by the microbiota varies and may be influenced by the specific composition of the gut microbiota and the type of food consumed. The reduction of sulfate and the breakdown of cysteine can lead to the formation of H_2_S, which significantly affects the health of the digestive tract [132].

H_2_S can be produced in the absence of enzymes using glucose, GSH, organic and inorganic polysulfides, and elemental sulfur. H_2_S can be formed non-enzymatically by the reduction of elemental sulfur using NADPH or by the oxidation of glucose via the phosphogluconate pathway [133].

Oxidants in the human body can metabolize H_2_S, which acts as a reducing agent. ROS and reactive nitrogen species (RNS) are combated in the human body [134], and activated antioxidant enzymes reduce free radical reactions and thus protect against the effects of aging [135].

At a pH value of 7.4, H_2_S is mainly present in the form of the hydrogen sulfide ion (HS^−^) and the sulfide ion (S^2−^). A comparison of the undissociated H_2_S molecule with its dissociated forms shows that the dissociated versions are more stable at this pH value. The stability is influenced by the pH of the solution and the concentration of H_2_S in its related states. As the pH increases, the proportion of undissociated hydrogen sulfide decreases. At a pH of 7.4, about 80% of the H_2_S is present as HS^−^ and S^−^, while only 20% of the H_2_S molecule remains undissociated. The distribution of the different forms of H_2_S is crucial for its biological functions, as each form can interact with different cellular components and signaling pathways [136].

Intact H_2_S molecules can penetrate cell membranes more easily and interact with intracellular components. H_2_S can also act as a signaling molecule by activating specific receptors and signaling pathways. Conversely, the dissociated HS⁻ and S^2^⁻ ions are more stable and can participate in redox reactions and enzymatic processes in the cells [137].

**Figure 8:**
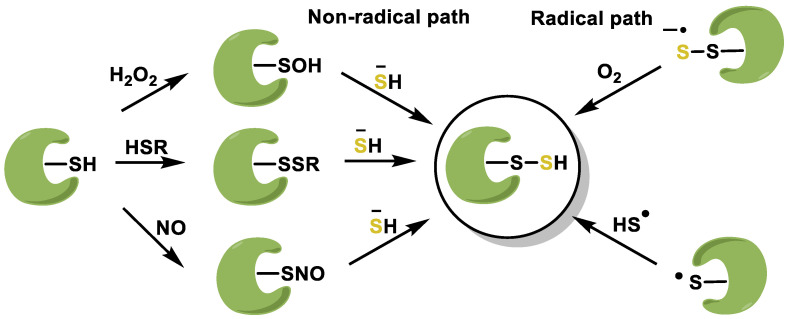
Proposed reaction mechanisms for persulphide formation. H_2_S can react with sulphenic acids. The reaction of S-nitrosated cysteines with H_2_S leads to the formation of protein persulphides in this reaction. H_2_S could react with existing intermolecular or intramolecular disulphides, while the sulphane sulphur in the polysulphides could react directly with protein thiols and give persulphide. HS^•^ radicals can be generated by H_2_S through oxidation by metal centers. HS^•^ will then react with O_2_ to generate protein persulfidation and HO_2_^•^.

Sulfhydration, also known as persulfidation, is a post-translational modification (PTM) that occurs in proteins and affects cysteine residues [138]. The formation of a thioether bond involves the linkage of a sulfhydryl group (-SH) from a cysteine residue to the sulfur atom of another molecule. This change can have a significant effect on the structure, stability, and function of proteins. Persulfidation of proteins is a post-translational modification (PTM) induced by H_2_S and its derivatives [139]. Persulfides can be generated by the reaction of HS^•−^ with RS^−^ using radicals. The radical anion RSSH^•−^ is formed by the interaction of HS^•−^ with a non-radical thiol (Figure 8).

H_2_S acts as a messenger molecule by sulfhydrogenating cysteine residues in proteins such as Keap1 in the process of protein nitrosylation [140]. H_2_S is responsible for the sulfhydrogenation of several proteins involved in different signaling cascades. This leads to changes in cell signaling pathways associated with oxidative stress, inflammation, cell survival, apoptosis, and cell metabolism [141]. S-sulfhydration is a process in which H_2_S interacts directly with the NRF2 signaling pathway. Sulfation of the Keap1 protein occurs at cysteine-151 residues 226 and 613, hindering the interaction between the newly formed NRF2 and the Keap1 protein, allowing the Keap1 protein to enter the nucleus to activate antioxidant genes by binding to ARE sites [142].

H_2_S appears to be responsible for the post-translational modification of the protein that modulates Keap1 activity. These post-translational modifications may affect the ability of Keap1 to interact with NRF2, possibly allowing for its translocation to the nucleus [37].

### 5.1. Availability of H_2_S in Age-Related Diseases and Aging

H_2_S has several biological effects, including antioxidant, anti-apoptotic, pro-angiogenic, and vasodilatory effects and enhancing the activity of endothelial NO synthase (eNOS), which regulates eNOS function [143]. H_2_S is crucial for the modulation and regulation of several signaling pathways in mammalian organisms that play a role in metabolic processes, cardiac function, and cell survival [144]. The cardiovascular system, blood vessels, and blood components are severely affected by its presence [145], having an impact on mitochondrial function and metabolic activities of the cell [146].

Low levels of H_2_S are associated with many cardiovascular diseases, including hypertension, atherosclerosis, and damage to heart tissue [145]. Research suggests that older adults with hypertension tend to have lower levels of endogenous H_2_S, suggesting that H_2_S plays a critical role in the regulation of blood pressure [147]. H_2_S and nitric oxide (NO) both cause vasodilation and are linked to blood pressure regulation.

H_2_S supplementation has shown promise in the treatment of cardiovascular problems such as ischemia-reperfusion injury to the heart muscle [148].

Diabetes is most commonly responsible for cardiovascular disease, blindness, kidney failure, and lower limb amputations. Research has shown that hydrogen peroxide (H_2_S) can control the metabolism of cellular energy sources, reduce insulin resistance, protect cardiomyocytes, and improve cardiac function by affecting key proteins such as differentiation cluster 36 (CD36) and glucose transporter 4 (GLUT4) [149].

Eugenia Piragine and colleagues conducted a systematic review and meta-analysis in 2023 to investigate H_2_S levels in patients with age-related diseases. The main finding was that patients with vascular or renal diseases, such as hypertension, showed a remarkable decrease in H_2_S levels compared with the control group, indicating lower circulating H_2_S levels in individuals with chronic and degenerative diseases compared with healthy individuals [150].

H_2_S has been shown to fight ROS and thus counteract cell aging [151]. There is evidence that H_2_S has a fundamental impact on the physiology and pathology of all species and tissues. The question of whether we can develop strategies for anti-aging treatments is likely to be raised [152].

### 5.2. Can H_2_S Donors Induce the H_2_S-Producing Enzymes CBS, CSE, and 3-MST?

This is a relevant question for a topic that is currently under investigation. At present, there are no significant reports showing a positive response to the question. Huerta de la Cruz et al. (2022) conducted a comprehensive analysis and argued that exogenous H_2_S can restore the expression of CSE and CBS, but not 3-MST. Their research found that the expression of CBS and CSE is reduced in the hypothalamus and brainstem after a severe traumatic brain injury. The research shows that daily injections of NaHS, an H_2_S donor, increase the production of CBS and CSE in a way that varies over time and by tissue. Nevertheless, these injections had no impact on 3-MST, suggesting that H_2_S production may be involved in abnormalities of the hypothalamus and brainstem after a traumatic brain injury [153].

The cystathionine β-synthase gene is located on chromosome 21q22.3 [154], and the cystathionine γ-lyase gene is located on chromosome 1p31.1 [155]. Since none of these genes are in the ARE region, it is not logical to expect a positive answer to the question at the head of this section.

### 5.3. Electrophilic Sulfur Compounds in the Human Diet

The human body can convert sulfur compounds present in foods such as garlic, onions, mushrooms, edible legumes, and fruits into H_2_S through chemical or enzymatic processes. The compounds obtained from natural sources are polysulphides substituted with allyl radicals. Garlic compounds such as diallyl sulfide, diallyl disulphide, and triallyl disulphide can generate H_2_S in the presence of glutathione [156].

Allicin is the main bioactive compound in freshly pressed garlic and some other Allium species produced by the chemical synthesis of a garlic clove. Despite its instability, this molecule has bioactive properties and exhibits several physiological functions, such as anticancer activity, modulation of the gut microbiota, antioxidant properties, and anti-inflammatory properties. Garlic has been shown to have a positive effect on cardiovascular health by lowering blood pressure, cholesterol levels, and platelet aggregation [157].

The chemical composition of garlic juice obtained by crushing garlic cloves differs significantly from that of whole garlic cloves, especially in terms of the number of organosulfur compounds present. Untreated garlic consists mainly of sulfur compounds [158].

**Figure 9:**
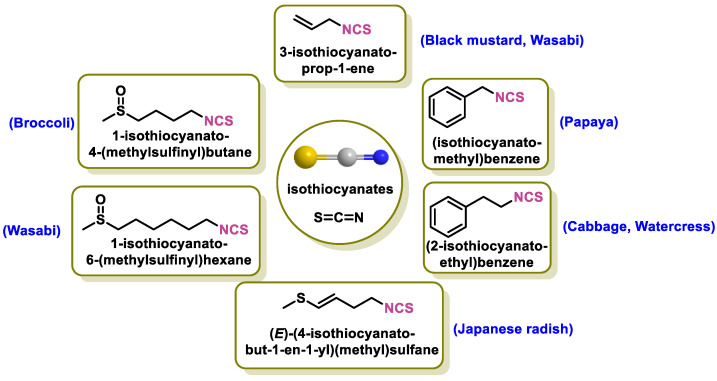
Examples of ITCs found in some foods.

ITCs are sulfur chemicals that occur naturally in plants. These compounds are characterized by the presence of a functional group known as –N=C=S. Cruciferous vegetables such as broccoli, watercress, Brussel sprouts, cabbage, and cauliflower contain ITCs, which are ECs [159] (Figure 9).

Glucosinolates (GSs) are the storage form of ITCs in plants and are released when the plant tissue is damaged. More than 120 different glucosinolates can be present in different plants, each of which provides a specific set of metabolites [160]. Broccoli contains a considerable amount of sulforaphane in the form of glucoraphanin [161]. The basic structure of a GS consists of an A–D thioglucose group, an oxime sulfonate group, and a variable side chain. The chemopreventive properties of cruciferous vegetables may be attributed to their high GS content. These glucosinolates are responsible for the strong odor and intense flavor that these plants possess [162].

The glucosinolates contained in cruciferous vegetables are converted by hydrolysis into ITCs, which are reactive organosulfur phytochemicals. These compounds have a unique and peculiar chemical reactivity and have anticancer, anti-inflammatory, and neuroprotective properties. In addition to their antibacterial effects, they can also reduce oxidative stress and act as indirect antioxidants [163].

ITCs exert various antimicrobial effects by reducing the O_2_ consumption of bacterial cells and depolarizing the mitochondrial membrane. As they are generally recognized as safe (GRAS), these chemicals may be used in food for preservation purposes [164].

Methionine serves as an intermediate in the synthesis of cysteine, carnitine, taurine, lecithin, phosphatidylcholine, and other phospholipids [165]. Disturbances in methionine conversion are associated with the occurrence of atherosclerosis [166]. Ethylene, nicotianamine, salinosporamides, and several glucosinolates, such as glucokechiroline, glucoerucine, glucoiberine, glucoiberverine, glucorraphanine, and sulforaphane, depend on methionine as it is a crucial element in their production [167,168]. Kachungwa Lugata et al., 2022, studied how methionine supplementation affects OS status in poultry. L-Met has been shown to increase the antioxidant capacity of poultry by increasing the levels of reduced GSH, superoxide dismutase (SOD), catalase (CAT), glutathione peroxidase (GPX), and other antioxidant enzymes [169]. This is supported by tests showing that it increases antioxidant levels and reduces oxidative stress.

### 5.4. Dithiolethiones Are Sulfur Compounds

**Figure 10:**
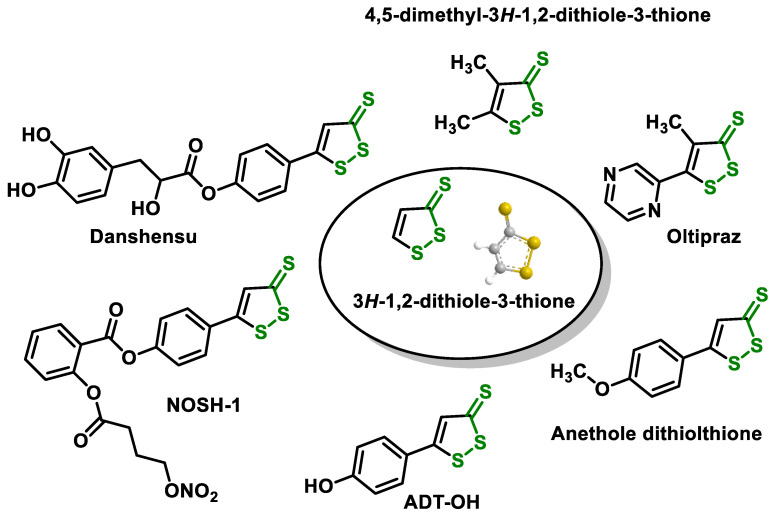
Drugs containing the 1,2-dithiole-3-thione moiety.

3H-1,2-dithiole-3-thione (D3T), a basic cyclic sulfur-containing compound, is found in small amounts in cruciferous vegetables. This compound is the simplest of the cyclic sulphur-containing dithiolethiones. D3T can boost the production of GSH and antioxidant genes by affecting the NRF2 [170,171,172] and promotes the dissociation of Keap1 from NRF2, leading to a rapid accumulation of NRF2 in the cytoplasm [173,174]. In addition to its antioxidant effects, D3T has been shown to affect inflammatory diseases such as endotoxemic shock, multiple sclerosis (MS), and light-induced retinal damage in animal models [175,176,177]. D3T, which is based on the NRF2 defense pathway, has been proposed as a potential treatment for ischemic stroke [178]. The D3T moiety is present in commercial drugs such as Oltipraz, Anethole dithiolethione, S-Danshensu, and NOSH-1 [179] (Figure 10).

There is a growing interest in compounds containing the 1,2-dithiol-3-thione moiety because of its ability to independently produce H_2_S.

### 5.5. Sulfurous Waters in Spas

**Figure 11:**
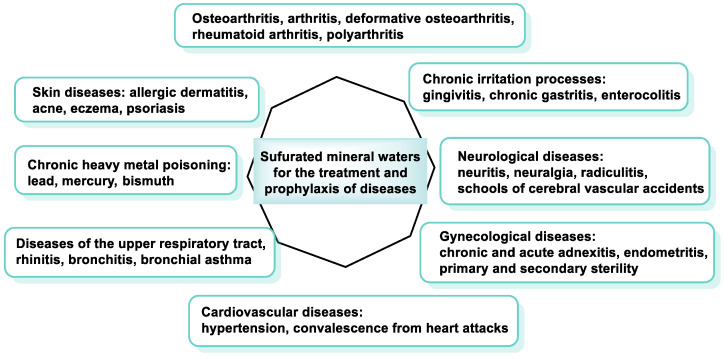
Examples of benefits of balneotherapy.

Balneotherapy is the use of thermal or mineral waters for therapeutic purposes. It has a long history and been proven to be effective. Sulfur-containing liquids, which contain dissolved sulfur compounds, are of great benefit due to their therapeutic properties [180]. They have the potential to be beneficial for various health problems, such as musculoskeletal conditions (to relieve inflammation, pain, and stiffness in joints and muscles), skin conditions (to treat acne, eczema, and psoriasis), and respiratory conditions (to treat congestion and sinus problems) [181] (Figure 11).

Pelotherapy is the name given to the use of mud or peat baths for medicinal purposes. Peloids are naturally occurring deposits of clay or mud that contain beneficial minerals and chemicals for the body. Pelotherapy with sulfurous peloids combines the benefits of balneotherapy with the benefits of the peloids themselves [182].

Research has shown that the combination of pelotherapy and balneotherapy in sulfurous water is more effective. This is probably due to the impact that the different treatments have on each other [183].

Sulfur-containing fluids have the potential to be effective in the treatment of osteoarthritis and osteoarthritis (OA). The sulfur compounds in sulfur-containing waters can reduce inflammation and relieve pain. They can also improve joint lubrication and relieve discomfort [184].

One study found that people with osteoarthritis experienced a significant reduction in pain and stiffness and improved function when they underwent balneotherapy and pelotherapy and consumed water compared with untreated patients [185]. Another study showed that patients with knee osteoarthritis who drank sulfur-containing water for four weeks experienced a significant decrease in pain and an improvement in function compared with those who drank tap water [186].

## 6. Role of Michael Acceptors

### 6.1. Polyphenols

Phenolic chemicals, sometimes referred to as polyphenols, are a diverse group of secondary plant metabolites. These chemicals are important components of plant defense mechanisms. Due to their high concentrations in foods such as fruits, vegetables, nuts, seeds, and spices, they are crucial to our diet [187].

**Figure 12:**
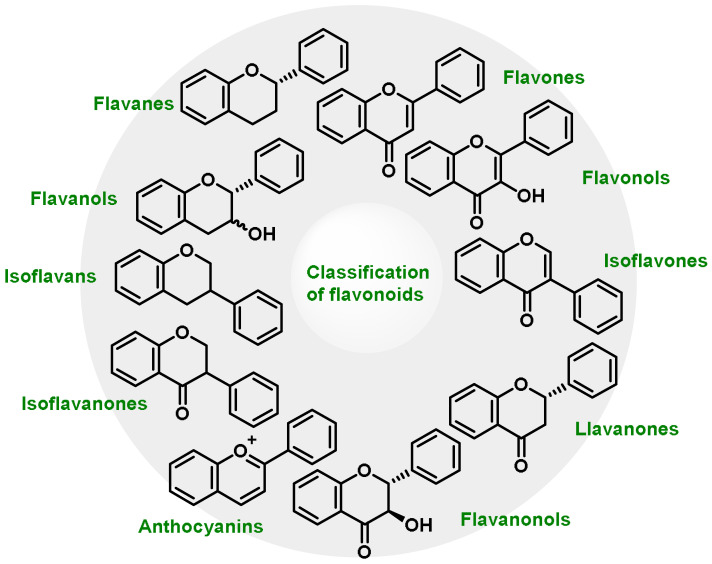
Structure of flavonoid families.

Phenolic compounds differ from other compounds by the presence of one or more aromatic rings containing hydroxyl groups. Phenolic molecules have unique properties such as antioxidant, anti-inflammatory and antibacterial properties [188]. The most common types of phenolic compounds include phenolic acids (the most prevalent group, characterized by the presence of a carboxyl group (-COOH) on the aromatic ring, such as gallic acid, hydroxycinnamic acids (caffeic, chlorogenic, and ferulic acid), and benzoic acid), flavonoids (which are classified into 12 major subclasses based on chemical structures, such as anthocyanidins, flavan-3-ols, flavonols, flavones, flavanones, and isoflavones, that have dietary significance) (Figure 12), lignans, a group of phenolic compounds derived from phenylpropanoid metabolism (secoisolariciresinol, lariciresinol, and pinoresinol), and others including stilbenes (e.g., resveratrol), anthocyanins (which give fruits their red, purple, and blue colors), and coumarins.

**Figure 13:**
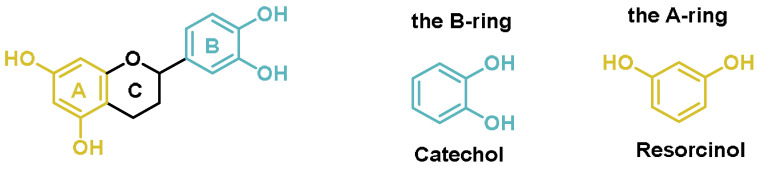
Catechol and resorcinol groups.

As antioxidants, polyphenols can counteract free radicals and reactive species, i.e., unstable chemicals that can damage various molecules in cells, such as proteins [140]. Exposure to environmental factors such as ultraviolet radiation, tobacco smoke, and pollution leads to the formation of free radicals in the body [189]. Polyphenols can protect cells from the damaging effects of free radicals, reducing the risk of chronic diseases such as diabetes, heart disease, and cancer. Polyphenols have several biological properties, such as antioxidant properties, that are largely responsible for their activities. In polyphenols, catechol is an organic functional group consisting of a benzene ring with two hydroxyl (-OH) groups attached to adjacent atoms of the ring (in the ortho position) and is typically found on the B-ring [190]. This functional group is responsible for many of the biological properties of polyphenols, including their antioxidant properties. The resorcinol group is typically found in the A-ring and is characterized by the two hydroxyl groups in the meta position [191] (Figure 13). Resorcinol and catechol are nucleophilic intermediates used in synthesis [192].

Phenols and polyphenols are nucleophiles due to the high electron density on the oxygen atom of the hydroxyl group and this makes them susceptible to electrophilic substitution reactions [193,194]. In these reactions, an electrophile, which is an electron-deficient species, is attracted to the electron-rich nucleophile and forms a bond with it.

**Figure 14:**
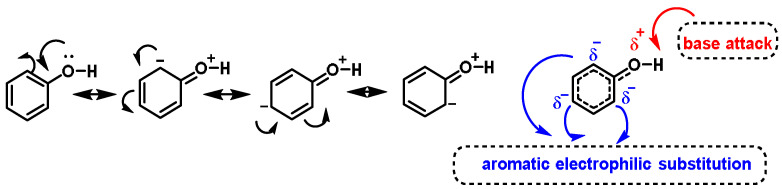
Resonant forms of phenol.

The phenolic -OH group delocalizes the charge in the benzene ring through resonance because the lone pair of electrons on the oxygen atom of the hydroxyl group can participate in resonance with the aromatic ring, spreading the charge over the entire molecule and increasing its stability [195]. This is a key characteristic of phenols and polyphenols and contributes to their unique chemical behavior (Figure 14).

Oxidative enzymes such as cytochrome P450 (CYP), cyclooxygenase-2 (COX-2), peroxidase, tyrosinase (monophenol oxygenase), xanthine oxidase (XO), monoamine oxidase (MAO), and polyphenol oxidases (PPOs) catalyze the oxidation of the catechol group in polyphenols [196]. As a result of this process, extremely reactive O-quinones are formed, which can react with cysteine residues in proteins or the sulfhydryl group of cysteine in GSH [197].

Polyphenol oxidases (PPOs) are indeed widely distributed in the animal and plant world. They catalyze the following two types of reactions [198]:

- The o-hydroxylation of monophenols to o-diphenols (catechols), also known as monophenolase activity;
- The oxidation of o-diphenols to o-quinones, also known as diphenolase activity.

**Figure 15:**
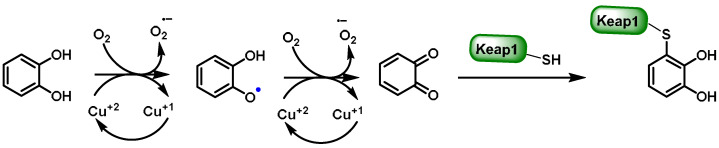
Mechanism of oxidation of the catechol group to o-quinones.

The o-quinones formed are very reactive and can enter into further chemical reactions. These interactions can lead to the production of dark pigments, melanins, which are primarily responsible for the brown coloration of fruit and vegetables [196]. Various oxidase and reductase enzymes ensure a redox balance when the catechol group is oxidized to o-quinone [199]. This equilibrium enables the reversible conversion of polyphenolic groups into quinones through the formation of reducing species such as H+ and e− [54] (Figure 15). Thus, maintaining an appropriate balance between nucleophilic chemicals and electrophilic substances is crucial for the activation of the NRF2–ARE axis, the master regulation of redox responses, and detoxifying signaling components [200].

**Figure 16:**
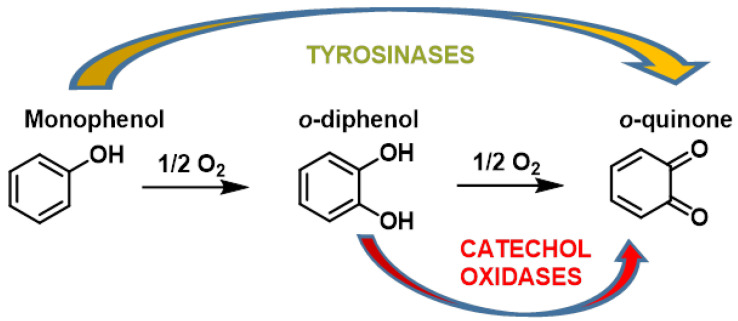
Oxidation of phenols to o-quinones catalyzed by PPO and related enzymes.

The hydroxylation reaction proceeds more slowly than the oxidation reaction. Quinones are formed by this mechanism [198,199,200,201] (Figure 16).

Understanding the dual role of polyphenols as antioxidants and pro-oxidants can help develop specific therapies to support advances in research and medicine [54].

### 6.2. Quinones

Quinones are organic molecules that occur naturally as colorants in plants and animals. Quinones are cyclic organic compounds with two ketone groups that occur frequently in nature [202]. One of the 1,4-benzoquinone derivatives is a constituent of ubiquinone or coenzyme Q, which is present in all living organisms [203]. One of the derivatives of 1,4-benzoquinone is a component of ubiquinone. This coenzyme is involved in the function of the electron transport chain in the inner membrane of the mitochondria. It is also involved in oxidative phosphorylation, the mechanism responsible for the production of ATP, the main source of energy for living organisms [204].

**Figure 17:**
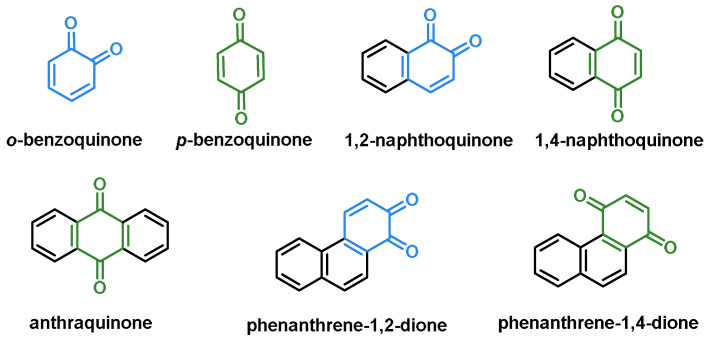
Quinone classification.

Quinones are also present in many herbs traditionally used, such as rhubarb, cassia, senna, comfrey, giant knotweed, polygonum, and aloe vera [205]. Quinones, which contain hydroxyl groups as auxochromes, exhibit a variety of hues, such as yellow, orange, reddish brown, and purple [202], (Figure 17).

**Figure 18:**
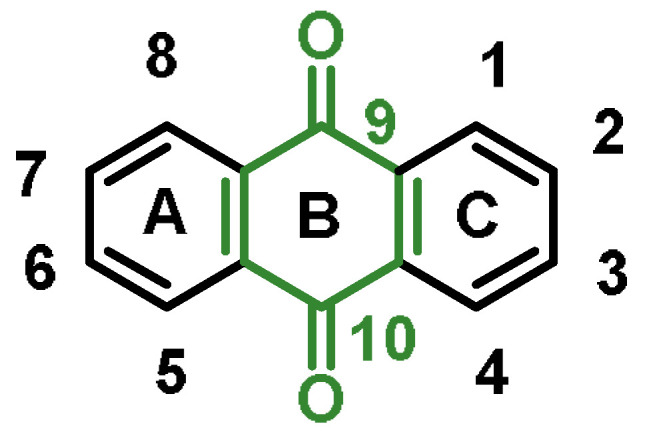
Molecular structure of anthraquinones.

Anthraquinones, (Figure 18), a type of quinonoid, are found in various foods, such as plants, fermented products, insects, and similar objects. The uses of anthraquinones, such as colorants, antioxidants, and antibacterials, are often determined by empirical knowledge. This structure is associated with various adverse properties, such as being a potential carcinogen [206].

The 9,10-anthraquinones, also known as 9,10-dioxoanthracenes, are an important subclass. They are built around a rigid, planar anthracene system with three aromatic rings (A, B, and C) with two keto functions in positions 9 and 10 [207]. Natural anthraquinone derivatives differ in their substituents and the substitution pattern of the A and C rings.

Anthraquinones are versatile compounds known for their diverse applications, including use in dyes and pigments, such as in the production of the red dye alizarin [208], use in medicine, including derivatives such as anthracenediones and the anthracycline family of chemotherapeutics [207], use in papermaking, with 9,10-anthraquinone serving as a fermenter additive in the production of alkaline paper pulp [209], use in hydrogen peroxide production (a large industrial application of anthraquinones is for the production of H_2_O_2_) [210], use as a bird repellent on seeds [211], and use as an electrolyte in flow batteries for long-term electrical storage [212].

These applications are closely related to the properties they have, such as colorant, antioxidant, and antibacterial properties. It is important to note that, while some applications are based on empirical data, many of them are also backed by scientific research and evidence [213].

Aloe vera leaves and rhubarb roots and rhizomes contain aloe emodin, an isomer of emodin also known as 1,8-dihydroxy-3-hydroxymethylanthraquinone. This anthraquinone is contained in aloe latex, a secretion of the aloe plant. It has also been shown to have antibacterial properties and a strong stimulant laxative effect [214]. This drug shows no anti-tumor activity against the various forms of benign or malignant tumors. Aloe emodin has no carcinogenic effect when administered topically. However, it can enhance the carcinogenic properties of certain types of radiation [215].

Anthraquinones have been shown to activate the NRF2 signaling pathway. Aloin, a notable anthraquinone found in Aloe species, has been shown to stimulate the NRF2/HO-1 defense system and shows promise in reducing inflammation and damage in cardiomyocytes [216].

It was also discovered that the glycosides anthraquinone and naphthopyrone contained in the seeds of Cassia obtusifolia have a hepatoprotective effect via NRF2-mediated HO-1 activation including MAPK regulation [217].

Anthraquinones can interact with the NRF2 signaling pathway as ECs. This compound can trigger antioxidant responses and provide protection against oxidative stress in various cell types. The specific effects may vary depending on the type of anthraquinone and the environment of the biological system.

BTB and CNC homology 1 (BACH1), together with Keap1, is one of the negative regulators of NRF2, which in turn controls ARE-dependent gene expression. In contrast to catechins, the electrophilic nature of quinones is critical for the inhibition of BACH1 and activation of NRF2 by arylation of BACH1 [218]. This knowledge may help in the development of targeted drugs.

### 6.3. Chalcones

**Figure 19:**
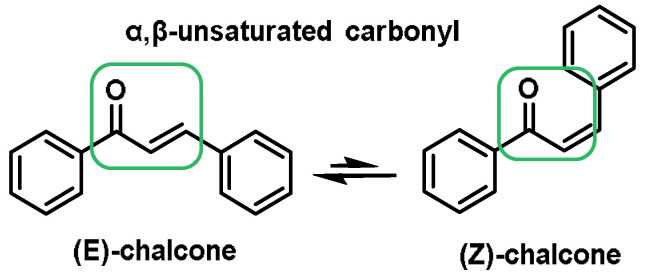
Trans and cis isomers of the basic chalcone skeleton.

Chalcones belong to the group of flavonoids. These flavonoids are open-chain flavonoids with three carbons between the two aromatic rings, which corresponds to their chemical composition. The carbons are linked by an α,β-unsaturated carbonyl system. Chalcone derivatives generally have the chemical structure 1,3-diaryl-2-propen-1-one, also known as benzalacetophenone and benzylideneacetophenone [219]. Chalcone derivatives come in two forms (trans and cis isomers). The trans isomer (E) is the more thermodynamically stable of the two (Figure 19) [219].

These compounds are known as open-chain flavonoids because they are the biogenetic precursors of the flavonoids and isoflavonoids commonly found in plants. Chalcones are unique because they do not contain the “C-ring” found in the basic structure of flavonoids. Open-chain flavonoids are an alternative name for these antioxidants.

Chalcones come from plants belonging to the *Leguminosae, Asteraceae,* and *Moraceae* families. They have been extensively studied due to their biological effects associated with minimal toxicity [220,221,222,223].

**Figure 20:**
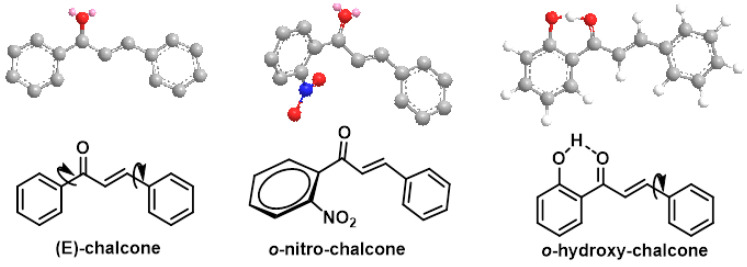
Molecular structure of chalcones.

Chalcones occur in a variety of natural compounds and are found in many plants such as vegetables, fruits, teas, and other natural products. Most naturally occurring chalcones exist as monomers, and their structural diversity results from the different types, amounts, and positions of substituents present. Bulky substituents in the ortho position, such as nitro or chlorine, change the planarity of the A or B ring and reduce the steric effects caused by these groups. The ring next to the carbonyl group is most susceptible to distortion [224]. Most natural and manufactured chalcones have a hydroxy substituent. When this group is in the ortho position, a strong intramolecular hydrogen bond is formed with the carbonyl oxygen, resulting in a stable planar conformation [225]. If the availability of the hydroxy group is reduced, this affects the reactivity and bioavailability of this group [110,226,227,228,229,230], (Figure 20).

**Figure 21:**
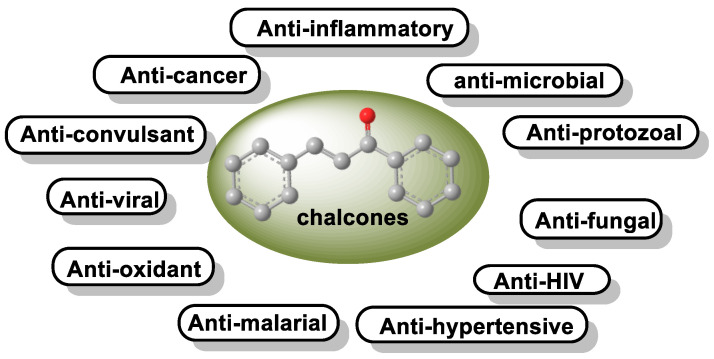
Biological activities of chalcones and their derivatives.

Plants that contain chalcones have been used in conventional medical practice for some time. In addition, a number of pharmacological studies have been approved for clinical trials for the treatment of cancer, viral diseases, and cardiovascular diseases, and chalcones have even been used as ingredients in cosmetic preparations [231] (Figure 21).

The incorporation of an α,β-unsaturated carbonyl group is primarily responsible for the biological activity of chalcones, which makes them Michael acceptor compounds [110,221,232]. To form the Michael adduct, this active component readily forms covalent bonds with nucleophiles such as the sulfhydryl group of cysteine residues in peptides or proteins in cells [232].

**Figure 22:**
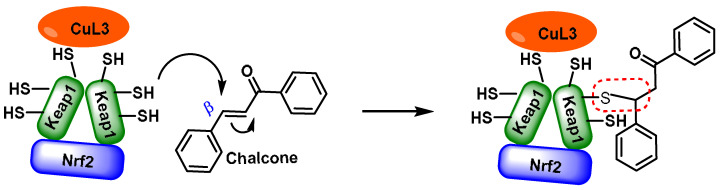
Michael’s addition of the Keap1 cysteine to the chalcone.

To obtain the Michael adduct, the α,β-unsaturated carbonyl molecule has the ability to rapidly form covalent bonds with nucleophiles, such as the sulfhydryl group of cysteine residues found in peptides or cellular proteins. Activation of the NRF2–ARE signaling pathway is a therapeutic target of chalcones. This activation occurs through the creation of covalent bonds with Keap1 cysteines and the carbon at the beta position of the α,β-unsaturated carbonyl residue [233] (Figure 22).

### 6.4. Curcuminoids

**Figure 23:**
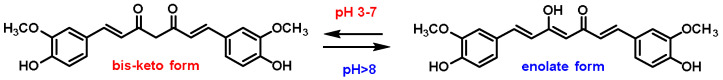
The two tautomers of curcumin. Those that are neutral or acidic are more likely to contain the bis-keto form, while those that are alkaline are more likely to contain the enolate form.

Turmeric is a spice native to India and Southeast Asia. Curcuminoids are a family of naturally occurring chemicals found in turmeric. One of the most well-known curcuminoid chemicals is curcumin (Figure 23), which is responsible for the striking yellow color of turmeric. Curcuminoids have been used in traditional Indian medicine since ancient times for their medicinal properties [234].

The properties of curcuminoids include the well-known antioxidant, neuroprotective, anti-tumor, anti-inflammatory, anti-acidogenic, radioprotective, and antiarthritic properties [108]. In addition to their use as additives, colorants, and spices, these compounds are also used in medicine as therapeutic agents. Curcuminoids have the potential to exert a therapeutic effect on a number of chronic diseases, such as colon cancer, lung cancer, breast cancer, and inflammatory bowel diseases, according to the preliminary results of a number of clinical trials [235].

**Figure 24:**
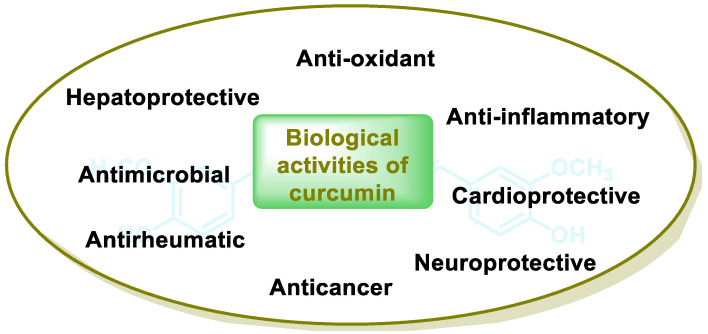
Several properties of curcuminoids.

Research has shown that curcuminoids have a wide range of potential health benefits. Firstly, they have antioxidant effects and protect cells from damage caused by free radicals, i.e., unstable molecules that can contribute to aging and disease [236]. Secondly, they are neuroprotective, protecting brain cells from damage caused by oxidative stress and inflammation, which are associated with neurodegenerative diseases such as Alzheimer’s disease and Parkinson’s disease [237]. Thirdly, they are anti-inflammatory and can reduce inflammation, which plays a role in many chronic diseases such as arthritis, cancer, and cardiovascular disease [238]. Fourthly, they have been shown to have anti-tumor properties in laboratory studies and some clinical trials are underway to evaluate their potential in the treatment of cancer. They are also anti-acidogenic and may help to reduce gastric acid secretion, which may be beneficial for people with heartburn or acid reflux [239]. Fifthly, they are radioprotective and can protect cells from damage caused by radiation, which may be beneficial for people undergoing radiation therapy for cancer [240]. Finally, they are antiarthritic and can reduce the inflammation and pain associated with arthritis [241], (Figure 24).

The NRF2 pathway is thought to be triggered by curcumin. Curcumin has two electrophilic α,β-unsaturated carbonyl residues capable of covalently binding to a cysteine residue of Keap1 [242].

### 6.5. Coumarins

**Figure 25:**
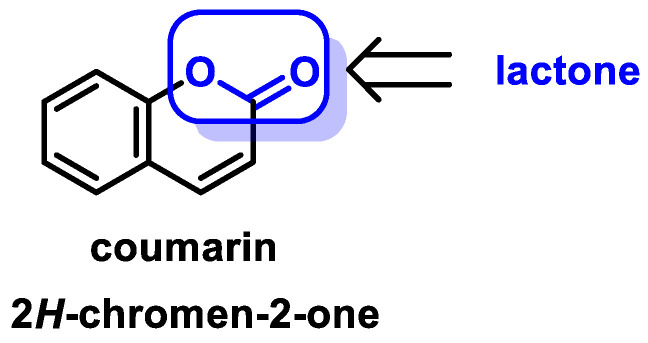
The structure of coumarin.

Coumarins, which are phenolic chemicals, are derived from the shikimic acid pathway. Lactones, regardless of whether they are of natural or synthetic origin, and their derivatives belong to a family of heterocyclic chemicals. These compounds have an electron-rich, conjugated π-π-π system and contain the 1-benzopyran-2-one backbone, also known as 2H-chromen-2-one. Coumarins have either a natural or synthetic origin [243] (Figure 25).

Plant food components commonly used in the human diet produce coumarin or its derivative compounds as metabolites derived from the fermentation process of the intestinal microbiota. These species belong to different botanical families (*Fabaceae, Rubiaceae, Rutaceae, Asteraceae, Umbeliferae, Apocinaceae, Compositae, Orquidaceae, Asteraceae, Umbeliferae, Apocinaceae, Compositae, Orchidaceae, Rutaceae,* and *Labiatae*).

Coumarin was first isolated by Vogel in 1820 from the seeds of the tonka bean, which belongs to *Dipteryx odorata* [244]. Tonka bean oil contains a high concentration of coumarin, which produces a pleasant fragrance reminiscent of vanilla. This secondary metabolite is used as a fragrance in both culinary and cosmetic products. Almost one thousand variants of this secondary metabolite have been isolated from more than eight hundred different species [245].

**Figure 26:**
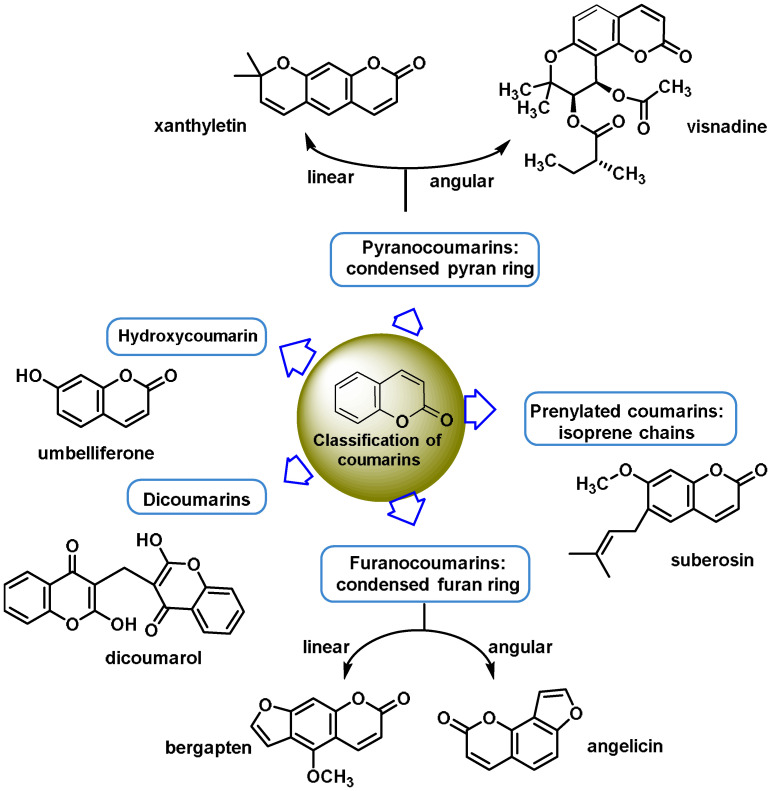
Classification of coumarins according to their chemical structure.

There is evidence that plants actually contain the glycosides of trans- and cis-ortho-coumaric acid and that coumarin is only released by enzymatic hydrolysis and lactonization when the plant tissue is damaged during harvest [246]. Although coumarin itself is found in plants, there is evidence that plants actually contain these glycosides. It is likely that coumarins are among the most numerous natural products, and they consist of a wide variety of chemicals (Figure 26).

**Figure 27:**
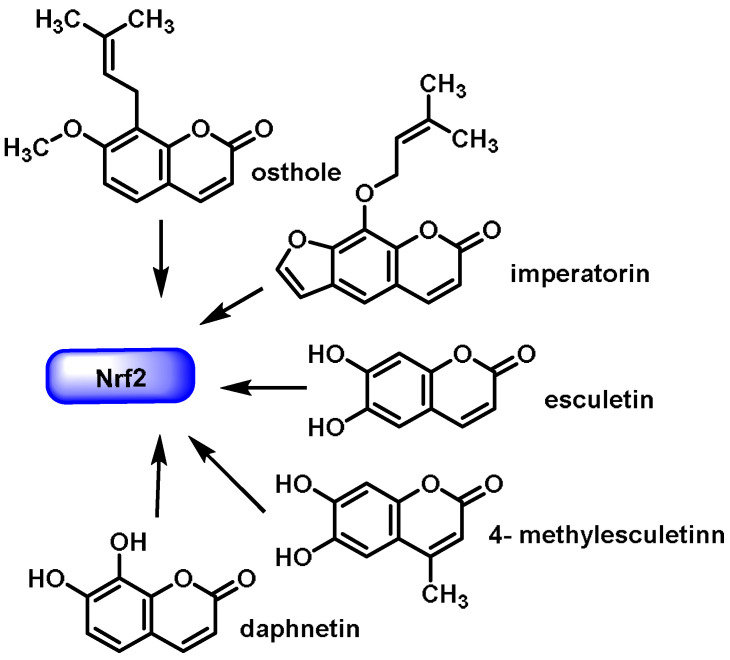
The chemical structures of coumarins that exert an anti-inflammatory effect on the intestine by regulating the NRF2 signaling pathway.

The coumarin ring is a component of a wide range of chemicals used in medicine. These substances include anticancer, antioxidant, antifungal, anticoagulant, anti-inflammatory, antiviral, antibacterial, antiprotozoal, and vasodilator molecules. In addition, it can inhibit enzymes such as cholinesterase, carbonic anhydrase, monoamine oxidase, serine protease, cyclooxygenase, and lipoxygenase [247,248,249,250,251,252,253,254,255,256,257,258]. In addition to the extraction of coumarins from hundreds of plant species and other microorganisms, there are also several derivatives of synthetic origin. These derivatives have considerably increased the number of known coumarin structures as well as the complexity of these structures [259]. It is plausible that stimulation of the NRF2 signaling pathway may provide an explanation for some of the pharmacological and biological effects of coumarins. Di Stasi et al., 2023 recently published a study that showed the ability of certain coumarins to activate the NRF2 signaling pathway in a variety of cell types and animal models. Coumarin derivatives such as esculetin, 4-methylsculetin, daphnetin, osthole, and imperatorin are recommended by these authors as potential lead compounds for the development and production of NRF2 activators that exhibit anti-inflammatory activity in the gut [260] (Figure 27).

Coumarin derivatives can either directly promote anti-inflammatory activity in the gut or indirectly induce pro-inflammatory activity through different mechanisms by reducing oxidative stress and regulating the NRF2 signaling pathway.

Coumarin can be coupled with other bioactive substances to form dimers and hybrids of coumarin, such as coumarin–chalcone, coumarin–imidazole, coumarin–pyrazole, coumarin–triazole, coumarin–benzotriazole, coumarin–isoxazole, coumarin–dihydroartemisinin, coumarin–hydrazine, coumarin–ergosterol, coumarin–ferrocene, coumarin–pyridine, coumarin–pyrimide, coumarin–benzosulfone, coumarin–imine, and coumarin–uracil hybrids, which have a broad spectrum of significant biological effects [253,254,255,256,257].

Urolithins are benzocoumarins derived from diphenylpyrran-6-one. Urolithin A and urolithin B reduce ROS generation, scavenge free radicals, and activate the NRF2 signaling pathway through nuclear translocation of NRF2 with the upregulation of HO-1, SOD, the GSH-related antioxidant system, and NQO1 [261].

### 6.6. Terpenoids

Terpenoids, including mono-, sesqui-, di-, and triterpenoids, are a large and diverse class of naturally occurring compounds. Terpenoids are found in the plant kingdom. One of the possible mechanisms of these terpenoids as inducers of NRF2 is their involvement as Michael acceptors in the reaction with the reactive cysteine residues of the Keap1 protein [262].

**Figure 28:**
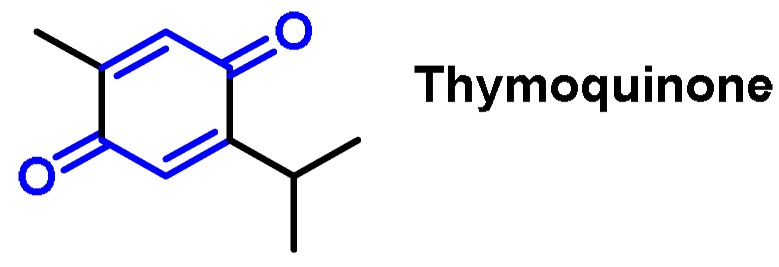
Chemical structure of thymoquinone.

Thymoquinone (2-isopropyl-5-methylbenzo-1,4-quinone) (Figure 28) is an active component belonging to the monoterpenoid isolated from *Nigella sativa* that possesses an alkylated benzoquinone structure.

Thymoquinone exhibits antioxidant and anti-inflammatory effects on various abnormalities associated with an oxidative stress imbalance and inflammation.

**Figure 29:**
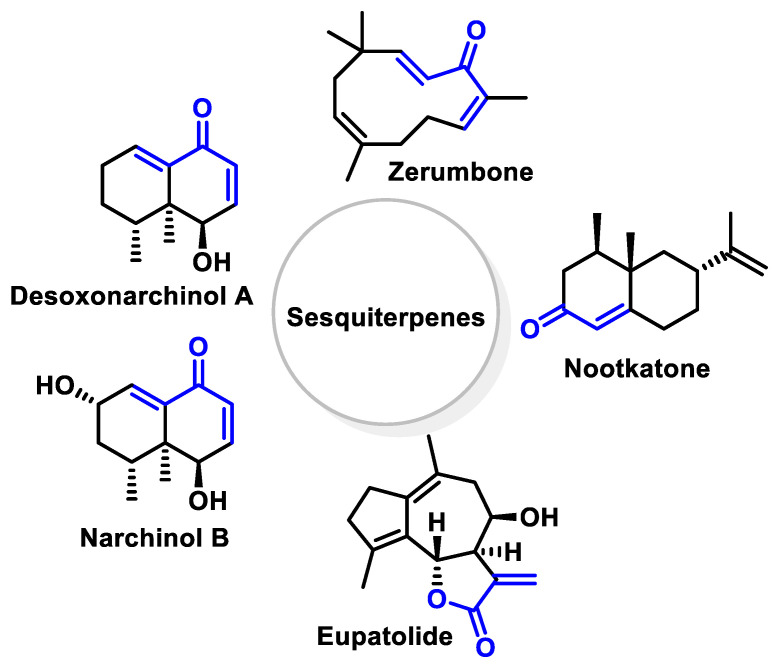
Chemical structure of some sesquiterpenes.

Among the sesquiterpenes (Figure 29), deoxonarchinol A isolated from *Nardostachys jatamansi* is an effective inducer of HO-1, which regulates neutrophil infiltration in acute pancreatitis by inhibiting chemokine ligand 2 [263]. Anti-neuroinflammatory effects through positive regulation of NRF2/HO-1 signaling by deoxonarchinol A together with narchinol B have also been investigated [264]. Nootkaton, extracted from Cyperus rotundus, is used in traditional medicine to treat inflammation. Its potential anti-inflammatory effect is thought to be related to the induction of HO-1, with the sesquiterpene nootkatone playing an important role in this mechanism [265]. *Inula britannica* contains a sesquiterpene lactone called Eu-patolide, which has the potential to inhibit platelet-derived, growth-factor-induced proliferation and migration of vascular smooth muscle cells. This occurs through the induction of HO-1 via the ROS–NRF2 pathway. In addition, this chemical has the potential to be an HO-1 inducer that can be used to prevent or treat vascular disease [266]. Zerumbone, also known as 2E, 6E, 10E-2,6,9,9-tetramethylcycloundeca-2,6,10-trien-1-one, is a monocyclic sesquiterpene extracted from the volatile essential oils of the rhizomes of *Zingiber zerumbet*.

**Figure 30:**
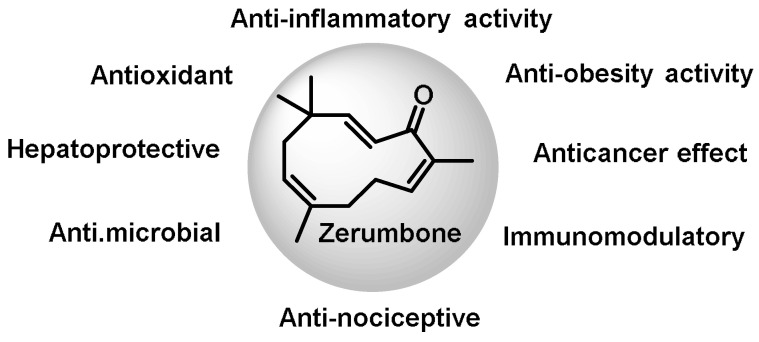
Pharmacological effects of zerumbone.

Although there have been positive preclinical studies demonstrating the therapeutic efficacy of Zerumbone, the clinical development of this drug has been hampered due to its low water solubility. Zerumbone is capable of producing a broad spectrum of biological and pharmacological effects, including the modulation of a variety of molecular targets and signaling systems [267] (Figure 30).

According to a 2017 study by Leung et al., the protective activities of Zerumbone in acute lung injury are thought to be mediated by the positive modulation of NRF2/HO-1 signaling [268].

Palbinone is a bioactive diterpene discovered in *Paeonia suffruticosa*. It has traditionally been used to invigorate blood circulation and to treat liver and inflammatory diseases. Recently, palbinone was found to stimulate HO-1 expression in liver cells. Palbinone belongs to the group of diterpenes that have Michael acceptor fragments in their structure [269]. *Isodon serra* was used to isolate oridonin, which was found to have an immunosuppressive effect [270]. Andrographolide is a diterpene identified from the oriental medicinal plant *Andrographis paniculata*. It has a five-membered unsaturated lactone unit.

**Figure 31:**
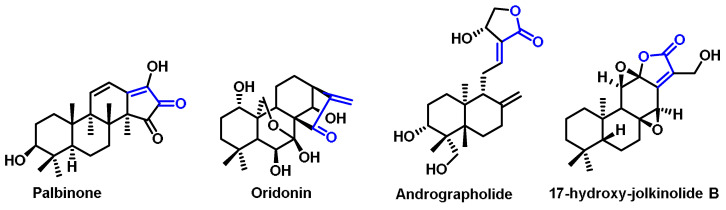
Chemical structure of some plant diterpenes.

Another diterpenoid with a five-membered lactone is called 17-hydroxyijolkinolide B and was identified from *Euphorbia fischeriana* (Figure 31).

17-hydroxyijolkinolide B can inhibit COX-2 and iNOS in a concentration-dependent manner. A reduction in MAPK phosphorylation, activation of NF-κB, and induction of HO-1 were found to be the components responsible for these inhibitory effects [271].

**Figure 32:**
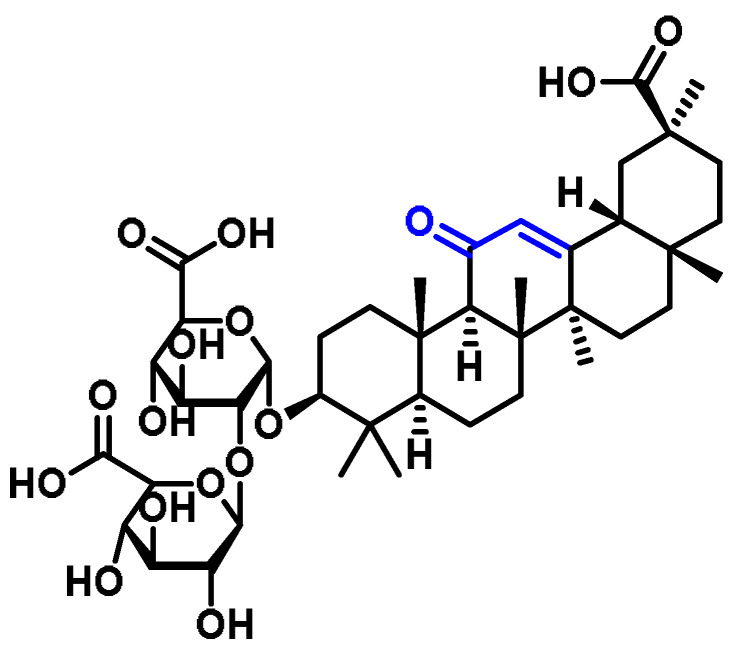
Chemical structure of glycyrrhizin.

Licorice, also known as *Glycyrrhiza glabra*, contains a triterpene glycoside called glycyrrhizin (Figure 32), which is responsible for the sweet taste and pharmacological activity of the plant [272].

Kim et al., 2015 proposed that glycyrrhizin generates p38/NRF2-dependent HO-1 induction, which may prevent sepsis [272]. Mou et al., 2019 demonstrated that glycyrrhizin protects human melanocytes from H_2_O_2_-induced oxidative damage through NRF2-dependent induction of HO-1 [273].

### 6.7. Steroids

**Figure 33:**
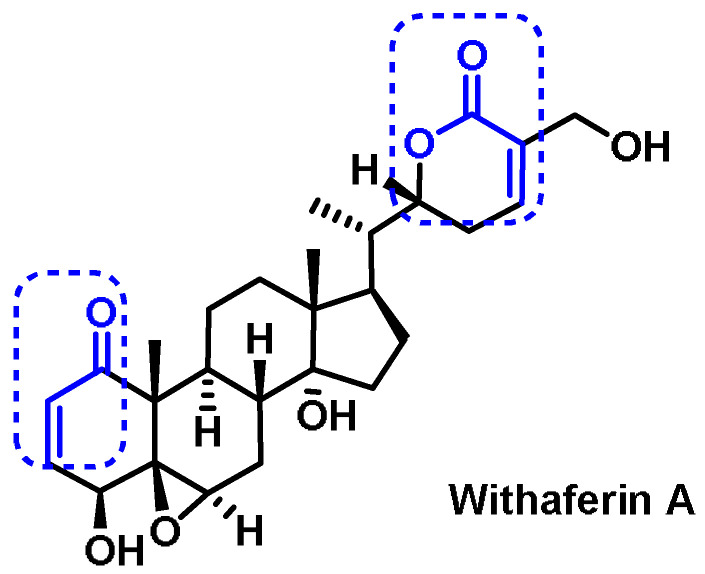
Chemical structure of Withaferin A.

An example of a steroid that possesses two Michael acceptors in its structure is Withaferin A (Figure 33). Withaferin A is a bioactive steroid phytochemical that is responsible for the bioactivities of *Withania somnifera*, also known as “Ashwagandha”, “Indian ginseng” or “winter cherry”, a medicinal herb often used in traditional Indian medicine [274].

Withaferin A induces HO-1 expression in endothelial cells through positive regulation and an increase in the nuclear translocation of NRF2 in a time- and concentration-dependent manner [274].

The NRF2–Keap1 pathway is activated by steroid hormone signaling in order to govern neuronal remodeling. Yuh Chew et al., 2021 demonstrated that the NRF2–Keap1 pathway, composed of CncC/NRF2, Keap1, and MafS, plays a cell-autonomous role in governing neuronal remodeling during Drosophila metamorphosis. NRF2–Keap1 signaling is activated downstream of the steroid hormone ecdysone. This study revealed an epistatic link between the NRF2–Keap1 pathway and steroid hormone signaling and demonstrated that the NRF2–Keap1 pathway plays an antioxidant-independent but proteasome-dependent role in neuronal remodeling [275].

### 6.8. Nitro Fatty Acids

**Figure 34:**
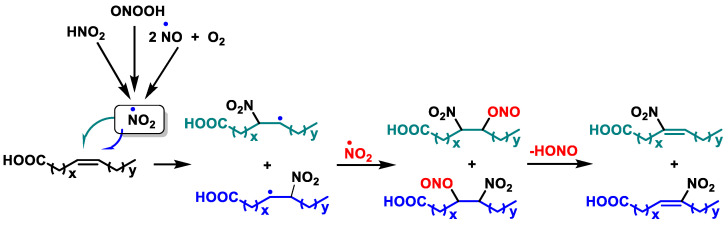
Endogenous nitration of UFAs.

Nitro-unsaturated fatty acids (NO_2_-FAs) can be formed endogenously by the reaction of unsaturated fatty acids (UFAs) with secondary nitrogen dioxide (NO_2_) species and nitrite anions [276] (Figure 34).

The NO_2_-FA moiety is a potent Michael acceptor that can react with the thiol-containing residues of biologically significant proteins, increasing the value of these molecules in terms of their therapeutic potential. The Mediterranean diet can be a source of NO_2_-FAs [277]. NO_2_-FAs reduce NF-κB and concurrently trigger the NRF2 pathway. NRF2 is activated by electrophilic fatty acids, suppresses redox-sensitive pro-inflammatory gene expression, and protects against vascular endothelial oxidative injury [278].

**Figure 35:**
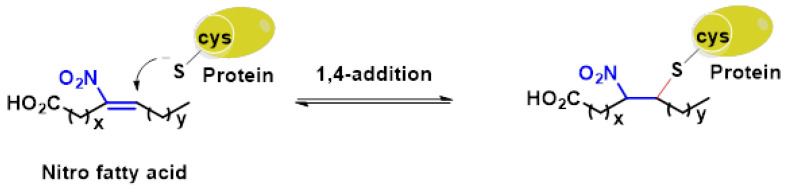
NO_2_-FA reaction with the thiol group of biologically relevant proteins.

NO_2_-FAs can be synthesized both endogenously in the body and exogenously in a laboratory. Stepwise synthesis requires specific precursor compounds and can provide NO_2_-FAs at a specific position [279]. In the case of NO_2_-FAs, the reaction is as shown in Figure 35.

NO_2_-FA-mediated effects contribute to symptom relief and actively enhance the resolution of inflammation by triggering the activation of the transcriptional factor peroxisome proliferator-activated receptor γ (PPAR-γ). The direct covalent binding of NO_2_-FAs to functionally significant amino acid residues of inflammatory goal proteins might help provide robust and constant pharmacological impacts [277].

### 6.9. Unsaturated Aldehydes (Cinnamaldehyde and Its Derivatives)

Cinnamaldehyde is an organic compound responsible for the characteristic taste and smell of cinnamon. It is a pale-yellow, viscous liquid that occurs naturally in the bark of the cinnamon tree and other species of the genus *Cinnamomum*. Cinnamon essential oil is 90% cinnamaldehyde [280].

**Figure 36:**
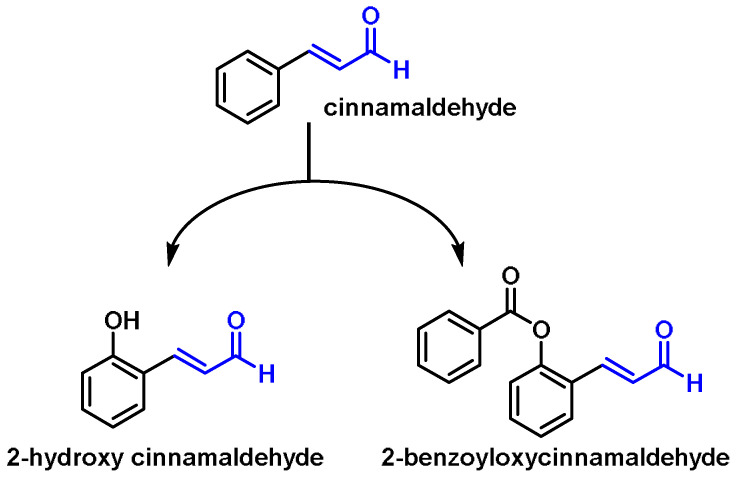
Chemical structure of cinnamaldehyde, 2-hydroxy cinnamaldehyde, and 2-benzoyloxycinnamaldehyde.

Cinnamaldehyde and its derivatives (Figure 36) are chemicals that can behave as Michael acceptors (as electrophiles), react with some enzymes and receptors with high-electron-density centres, and produce various therapeutically relevant pharmacological functions. Cinnamaldehyde is an α-β-unsaturated aldehyde that covalently binds with Keap1 thiol groups, releasing and stabilizing NRF2. Transcinnamaldehyde, 2-benzoyloxycinnamaldehyde (2-BCA), and 2-hydroxycinnamaldehyde (2-HCA) are representatives of this group and exhibit anticancer, anti-inflammatory, antidiabetic, and antifungal properties. Cinnamaldehyde has also been reported to be beneficial against neurological diseases, such as PD and AD.

According to Huang et al., 2011, cinnamaldehyde enhances the nuclear translocation of NRF2 and upregulates the expression of phase II detoxifying enzymes in HepG2 cells. In this study, the authors investigated the molecular signaling events mediated by cinnamaldehyde, observing that the ERK1/2, Akt, and JNK signaling pathways were activated, but not the p38 MAP kinase pathway, leading to the nuclear translocation of NRF2 and an increase in the expression of phase II enzymes [281].

## 7. Role of Electrophilic Compounds in Diseases

In recent decades, ECs have attracted much attention in the field of drugs that are able to interact covalently. Electrophiles have long been recognized as mediators in inflammatory processes and can even modulate the immune response by regulating metabolic networks, as they function as pleiotropic signaling mediators capable of reversibly reacting with nucleophilic biomolecules, especially reactive cysteines [282].

In recent years, the development of targeted covalent inhibitors has gained popularity worldwide. These inhibitors involve specific groups known as electrophilic warheads, which form irreversible bonds with nucleophilic amino acid residues in biological targets, such as proteins [283].

Covalent drug discovery aims to place an electrophilic moiety on the inhibitor. Upon binding to the target protein, this moiety undergoes an attack from a nucleophilic amino acid residue, resulting in the formation of an irreversible bond. These bonds are much stronger than typical reversible interactions [284].

Covalent drugs have the advantage of long-lasting activity due to their strong bonds with protein targets. They allow for a high degree of potency to be achieved in compounds of low molecular mass, along with other beneficial pharmaceutical properties [285]. The development of small-molecule drugs that covalently inhibit biological targets dates back to 1897 with the discovery of aspirin. Although aspirin has been on the drug market since the early 20th century, its mechanism of action was not revealed until the 1970s, when Roth et al., 1975 demonstrated that aspirin irreversibly inhibited cyclooxygenase-1 (COX-1), an enzyme that plays an instrumental role in prostaglandin biosynthesis [286]. Aspirin acetylates a serine residue at the active site and thereby inactivates COX-1.

Over the past two decades, targeted drugs have made rapid advances, leading to important strategies for cancer treatment. Examples include the β-lactam-containing antibiotic penicillin, the chemotherapeutic fluorouracil, and Osimertinib [283]. Despite the progress, challenges remain, including high reactivity and “off-target” toxicity. Researchers continue to explore innovative strategies for designing effective covalent drugs [287]. Covalent inhibition may also be an underused strategy for addressing challenging targets and previously considered “undruggable” modalities in human disease [288]. In summary, the world of covalent drug discovery is dynamic and promising, with electrophilic small molecules playing a crucial role in shaping the future of medicine.

Many natural products have been advocated as anticancer agents, essentially because they contain functional groups characterized by their chemical reactivity. A substantial fraction (~20%) of cytotoxic synthetic compounds containing Michael acceptor groups inhibit proteasome substrate processing and induce a cellular response characteristic of proteasome inhibition. Biochemical and structural analyses showed binding to and inhibition of the proteasome-associated cysteine deubiquitinase ubiquitin-specific peptidase 14 (USP14). These findings suggest that proteasome inhibition is a relatively common mode of action used by cytotoxic compounds containing Michael acceptor groups and help to explain previous reports on the antineoplastic effects of natural products containing such functional groups [289].

While electrophiles are crucial in chemical reactions, there are concerns about their use because they can be “promiscuous” inside the body by bonding with unintended targets, leading to potential side effects [290].

Understanding the balance between beneficial and harmful effects is essential for the safe use of electrophiles in therapeutic strategies. In summary, electrophiles are essential for various reactions, but their effects must be carefully considered in order to avoid unintended consequences [291].

In the realm of health and medicine, ECs have garnered a significant amount of interest due to their potential therapeutic applications. Some future directions related to electrophiles are as follows:

- Researchers are actively investigating how ECs interact with biological molecules. Unraveling these mechanisms will enhance our understanding of their effects on cellular processes and disease pathways [292];
- Targeted therapies. The development of ECs that selectively target specific proteins or pathways holds promise. By designing molecules that interact with specific cellular components, we can create more effective and safer drugs [287];
- Precision medicine. The tailoring of electrophilic therapies to individual patients based on their genetic makeup and disease profile is an exciting avenue. Personalized treatments could optimize efficacy while minimizing side effects [293];
- Some electrophiles exhibit antioxidant properties by activating cellular defense mechanisms. Exploring their potential in conditions like neurodegenerative diseases and cancer is an ongoing area of research [294];
- Innovations in drug delivery can enhance the bioavailability and tissue specificity of ECs. Nanoparticles, liposomes, and other carriers can improve their therapeutic impact [295];
- Combination therapies. The integration of ECs with existing drugs or other treatment modalities could lead to synergistic effects. Combinations may enhance efficacy and reduce resistance [296];
- Safety profiling. Addressing the promiscuity issue (where electrophiles bond with unintended targets) requires rigorous safety profiling. Predictive models and screening assays can help identify potential adverse effects [297];
- Neuroprotection. ECs may play a role in preserving neuronal health. Investigating their impact on neuroinflammation, oxidative stress, and neurodegenerative disorders is crucial [298];
- Metabolic disorders. Exploring electrophiles as regulators of metabolic pathways (e.g., glucose metabolism and lipid homeostasis) could yield novel therapeutic strategies for conditions like diabetes and obesity [299];
- Environmental exposure. Investigating the impact of ECs from environmental sources (e.g., air pollution and dietary components) on human health is an emerging field [300].

In summary, the study of ECs offers exciting prospects for advancing medicine, but careful research and innovative approaches are essential in order to harness their full potential.

## 8. Bioavailability and Metabolism of Electrophilic Compounds

Bioavailability refers to the percentage of an administered dose of a substance, such as a drug or nutrient, that reaches the systemic circulation. In other words, it represents the degree to and rate at which a substance (such as a drug) is absorbed into a living system or becomes available at the site of physiological activity [301]. Understanding bioavailability is crucial because it determines how effectively ECs can exert their health-promoting effects [302]. The benefit of the function of bioactive molecules in the body is closely related to their bioavailability after ingestion.

When assessing the health benefits of ECs, it is important to consider their bioavailability, which is influenced by factors such as enzyme interactions, reactivity with other foods and the solubility of ECs when glycated in the ingested source, such as polyphenols in fruits and vegetables) [302].

Plant antioxidants include compounds such as polyphenols and terpenoids, which have traditionally been of great interest due to their potential to prevent CVD, cancer, neurodegenerative disorders, diabetes, and other diseases [303].

EC absorption and bioavailability are crucial, but the bioavailability of ECs is often low due to interactions with absorption processes mediated by the liver, intestine, and microbiota. Aglycones like quercetin generally have poor water solubility, further limiting their bioavailability [304]. Several factors can influence the bioavailability of ECs, including the following:

- Interactions with food components. ECs can interact with other components in the food matrix (such as fibers, proteins, and lipids), affecting their absorption. In the food matrix, ECs can be linked to carbohydrates, organic acids, hemicellulose, and cellulose [305];
- Phase I and II metabolism in the liver. These processes can alter the chemical structure of ECs. Phase I reactions of drug metabolism involve the oxidation, reduction, or hydrolysis of the parent drug, resulting in its conversion to a more polar molecule. This phase yields a polar, water-soluble metabolite that is often still active. Many of the products in this phase can also become substrates for phase II reactions. Phase II reactions involve conjugation by the coupling of the drug or its metabolites to another molecule, such as a glucuronidation, acylation, sulfate, or glycine molecule. This phase yields a large polar metabolite by the addition of endogenous hydrophilic groups to form inactive water-soluble compounds that can be excreted by the body [305];
- Absorption in the small intestine, where most ECs are absorbed. However, their uptake can vary based on their chemical form;
- The gut microbiota can further metabolize ECs, impacting their bioavailability [306].

The quantities of bioactive compounds present in broccoli and the bioavailability of ITCs after ingesting broccoli may be affected, as the cooking procedures used with the vegetable may affect both values. Considering the bioactivity and the potential chemopreventive activity of ITCs, steaming treatments can be considered the most appropriate method of cooking to enhance the health-enhancing benefits of broccoli in the diet [307]. According to Orlando et al., 2022, both boiling and steaming significantly decreased the total ITC equivalent level in florets of broccoli. Steaming minimized the loss of ITCs (−23% compared with frozen florets) in contrast to boiling, which almost halved them (−48%) [307].

Allyl thiosulfinates are responsible for most of the known health benefits of crushed raw garlic. They are absent in garlic cloves, but can be rapidly produced from alliin when endogenous alliinase is activated by crushing the cloves [308].

Most of the known health benefits of crushed raw garlic derive from allyl thiosulfinates (75% allicin content). These are absent in garlic cloves but are rapidly produced from alliin when the cloves are crushed (as endogenous alliinase is activated) [309]. Allicin production is inhibited by heat or an acidic pH (less than 3.5) [310]. However, allicin is quite unstable and readily breaks down into other compounds. This makes it difficult to measure its bioavailability, which refers to the amount of a substance that enters the bloodstream and becomes available to the body. Studies suggest that the bioavailability of allicin from raw garlic can be as high as 70–90%, while cooked garlic and garlic powder have significantly lower bioavailability (around 18% or less) [309]. It is difficult to assess the average total EC intake in the human diet. However, it is simpler and more effective to study the daily dietary intake of polyphenols for a given diet containing fruits, vegetables, and derived beverages (wine, coffee, tea, etc.). It is estimated that phenolic acids represent approximately one third of the total intake and flavonoids represent the remaining two thirds [311]. The most abundant flavonoids in the diet are flavanols (consisting of monomers known as catechins, dimeric procyanidins, trimeric procyanidins, procyanidins, and tannin polymers), anthocyanins, and their derivatives [312].

The study of the uptake and distribution of polyphenols is crucial in order to understand the potential of ECs in the prevention of oxidative-stress-induced diseases and their bioactive effects in living organisms [313,314]. The main dietary sources of polyphenols are fruit, vegetables, dried legumes, cereals, and beverages (fruit juice, wine, tea, coffee, chocolate milk, and beer). The approximate total intake is ~1 g/day [311]. Foods high in polyphenols, as well as factors such as polymerization and conjugation with other compounds, increase the degree of molecular complexity, making absorption studies difficult [315]. The most important determinant of the health benefits associated with polyphenols is the extent of their consumption. Other factors influencing the efficacy of polyphenols are their bioavailability and their ability to reach certain tissues [316]. In order for a non-nutrient EC to be absorbed in the intestine, it must be able to pass through the intestinal barrier and become biologically accessible, i.e., be released from the food matrix [317]. Polyphenols have a low level of bioavailability after absorption as the body perceives them to be xenobiotics that are eventually excreted [318]. This is the main problem with their pharmaceutical use. Dietary polyphenols in food are absorbed slowly in the stomach and small intestine [319]. Consequently, the polyphenols that are not absorbed continue their journey to the large intestine, where they are hydrolyzed, demethylated, decarboxylated, dehydroxylated, and ring-cleaved by the microbiota [320]. After undergoing these processes, the microbial metabolites undergo phase II metabolism in the large intestine and liver. They then enter the bloodstream and have biological effects that extend to the peripheral organs [321]. Polyphenols and their metabolites that are not absorbed by the body are excreted in the stool, while the microbial metabolites that are absorbed are mainly excreted in the urine [322]. In an in vitro study on digestion in the duodenum, Bermudez-Soto discovered that anthocyanins, flavonols, and flavan-3-ols were unstable (decreases of 43, 26, and 19%, respectively) [323]. Recent studies on the bioavailability of polyphenols indicate that, even when administered in high concentrations, the amount of these chemicals that can be absorbed by the body is low [324]. Part of the reason for the limited bioavailability of some polyphenols may be their possible loss in the duodenum [319]. The bioavailability of polyphenols is influenced by their interactions during digestion [325]. The glycosylation of polyphenols determines the absorption pathway, with glycones being absorbed by active transport and aglycones by passive diffusion [326,327]. The liver is the main organ responsible for detoxifying the body, while the small intestine helps to convert xenobiotics into a form that can be easily excreted [328]. The detoxification system in the liver and enterocytes significantly transform most polyphenols after enteral absorption before they are excreted via bile, feces, and urine [317]. Glycosylated polyphenols are hydrophilic, which is why it was long thought that they could not pass through the membranes of enterocytes. However, recent research has shown that certain nanotransporters facilitate their passage [328]. The glycosidic components of polyphenol glycosides are degraded by lactase phlorizin hydrolase (LPH), an enzyme located at the brush border of enterocytes [329]. Polyphenol glycosides are transported across the cell membrane of enterocytes by the active sodium-dependent glucose transporter SGLT1. When they enter the enterocytes, they are hydrolyzed by cytosolic β-glucosidase (CBG) in order to remove the glycosidic moieties [317,330].

**Figure 37:**
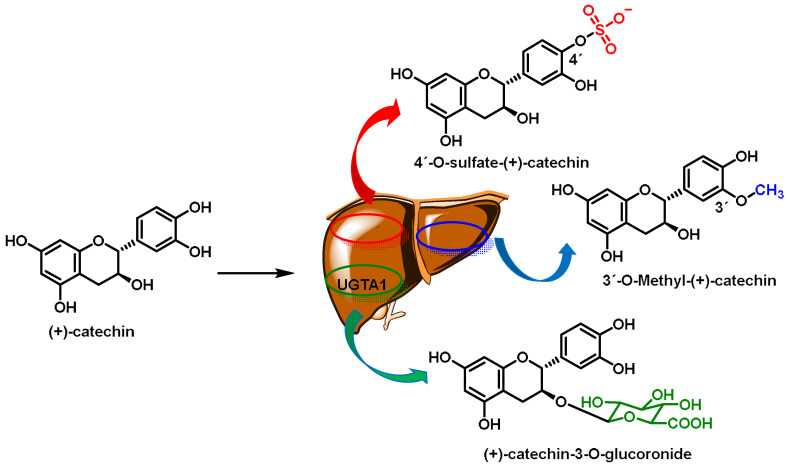
Hepatic enzymes in the metabolism of polyphenols using catechin as an example. The UGT enzyme, in the endoplasmic reticulum of hepatocytes, facilitates the conjugation of glucuronic acid. The SULT enzyme transfers a sulfate group from 3′-phosphoadenosine-5′-phosphosulfate to a hydroxyl group. COMT typically methylates hydroxyl groups at the catechol position of phenolic compounds, especially at the 3′-carbon of the B-ring.

After absorption in the small intestine, most of the aglycones are transported to the liver [331]. This process enables their rapid excretion and results in a short pharmacokinetic profile. In the liver, enzymes such as UDP-glucuronosyltransferases (e.g., UDPGT and UGT), sulfotransferase (P-PST and SULT), and catechol-O-methyltransferase (COMT) facilitate this process by conjugating hydroxyl groups with glucuronic acid, sulfate, or methyl groups [317,330] (Figure 37). In addition to rapid excretion, this conversion reduces the efficacy of the phytochemicals compared with their original aglycones [317]. The antioxidant activity of aglycones is reduced due to the loss of the hydroxyl groups responsible for hydrogen donation or electron transfer [331].

Finally, approximately 5–10% of ingested polyphenols reach the large intestine. Given their antimicrobial activity, polyphenols can also act as prebiotics by promoting the proliferation of beneficial bacteria, stabilizing the intestinal microbiota, and suppressing the growth of harmful bacteria [332,333]. In summary, the bioavailability and pharmacodynamic effects of the polyphenols and their metabolites depend on the interaction of transporter proteins, metabolic enzymes, intestinal bacteria, and host factors. Research is being conducted to fully investigate the elements that influence the bioavailability of polyphenols. These include the food matrix, co-consumed nutrients or meals, enzymes, the pH in the esophagus and colon, the microbiota in the colon, and the physicochemical properties of the polyphenols [318,334].

## 9. Conclusions

ECs are chemicals that can accept electrons from other molecules and can be found in various plants. ECs can react with biological molecules, such as proteins, lipids, and DNA. Among these reactions is the interaction with the Keap1 protein, which regulates the transcription factor NRF2, which in turn regulates the expression of genes involved in antioxidant defense. When activated, NRF2 induces the expression of genes coding for antioxidant enzymes such as catalase, glutathione peroxidase, and superoxide dismutase. These enzymes help protect cells from damage caused by ROS. The role that ECs and NRF2 play in human health is being increasingly recognized. It is known that NRF2 may be involved in the onset and development of various diseases, such as cancer, diabetes, cardiovascular disease, and neurodegenerative diseases. NRF2 has been proposed as a new therapeutic target for the treatment of these diseases.

NRF2 activates the expression of genes involved in the maintenance of the redox balance and other metabolic pathways, thus playing an important role in the development of various diseases. Further studies are needed to better understand the molecular mechanisms involved in this relationship and to evaluate the efficacy of NRF2 as a therapeutic target for the treatment of various diseases. In this review, we have attempted to link the consumption of certain ECs present in plants (such as polyphenols, ITCs, and garlic-derived compounds) with the inhibition of the Keap1 protein and the activation of NRF2.

## References

[1] Cardenas D. (2013). Let not thy food be confused with thy medicine: The Hippocratic misquotation. e-SPEN J..

[2] Who J., Consultation F.E. (2003). Diet, nutrition and the prevention of chronic diseases. World Health Organ. Tech. Rep. Ser..

[3] Diab A., Dastmalchi L.N., Gulati M., Michos E.D. (2023). A Heart-Healthy Diet for Cardiovascular Disease Prevention: Where Are We Now?. Vasc. Health Risk Manag..

[4] Tuso P.J., Ismail M.H., Ha B.P., Bartolotto C. (2013). Nutritional update for physicians: Plant-based diets. Perm. J..

[5] McMacken M., Shah S. (2017). A plant-based diet for the prevention and treatment of type 2 diabetes. J. Geriatr. Cardiol..

[6] Fardet A., Rock E. (2014). Toward a new philosophy of preventive nutrition: From a reductionist to a holistic paradigm to improve nutritional recommendations. Adv. Nutr..

[7] Sakanyan V. (2018). Reactive Chemicals and Electrophilic Stress in Cancer: A Minireview. High Throughput.

[8] Zaky A.A., Simal-Gandara J., Eun J.-B., Shim J.-H., Abd El-Aty A.M. (2022). Bioactivities, Applications, Safety, and Health Benefits of Bioactive Peptides From Food and By-Products: A Review. Front. Nutr..

[9] Gersch M., Kreuzer J., Sieber S.A. (2012). Electrophilic natural products and their biological targets. Nat. Prod. Rep..

[10] Yoo M.H., Xu X.M., Carlson B.A., Patterson A.D., Gladyshev V.N., Hatfield D.L. (2007). Targeting thioredoxin reductase 1 reduction in cancer cells inhibits self-sufficient growth and DNA replication. PLoS ONE.

[11] Iqbal I., Wilairatana P., Saqib F., Nasir B., Wahid M., Latif M.F., Iqbal A., Naz R., Mubarak M.S. (2023). Plant Polyphenols and Their Potential Benefits on Cardiovascular Health: A Review. Molecules.

[12] Summerhill V., Karagodin V., Grechko A., Myasoedova V., Orekhov A. (2018). Vasculoprotective Role of Olive Oil Compounds via Modulation of Oxidative Stress in Atherosclerosis. Front. Cardiovasc. Med..

[13] Moi P., Chan K., Asunis I., Cao A., Kan Y.W. (1994). Isolation of NF-E2-related factor 2 (Nrf2), a NF-E2-like basic leucine zipper transcriptional activator that binds to the tandem NF-E2/AP1 repeat of the beta-globin locus control region. Proc. Natl. Acad. Sci. USA.

[14] Li W., Kong A.N. (2009). Molecular mechanisms of Nrf2-mediated antioxidant response. Mol. Carcinog..

[15] Saha S., Buttari B., Panieri E., Profumo E., Saso L. (2020). An Overview of Nrf2 Signaling Pathway and Its Role in Inflammation. Molecules.

[16] Ngo V., Duennwald M.L. (2022). Nrf2 and Oxidative Stress: A General Overview of Mechanisms and Implications in Human Disease. Antioxidants.

[17] Taguchi K., Yamamoto M. (2020). The KEAP1-NRF2 System as a Molecular Target of Cancer Treatment. Cancers.

[18] Dodson M., de la Vega M.R., Cholanians A.B., Schmidlin C.J., Chapman E., Zhang D.D. (2019). Modulating NRF2 in Disease: Timing Is Everything. Annu. Rev. Pharmacol. Toxicol..

[19] Suzen S., Tucci P., Profumo E., Buttari B., Saso L. (2022). A Pivotal Role of Nrf2 in Neurodegenerative Disorders: A New Way for Therapeutic Strategies. Pharmaceuticals.

[20] Smith R.E., Tran K., Smith C.C., McDonald M., Shejwalkar P., Hara K. (2016). The Role of the Nrf2/ARE Antioxidant System in Preventing Cardiovascular Diseases. Diseases.

[21] Lewis K.N., Wason E., Edrey Y.H., Kristan D.M., Nevo E., Buffenstein R. (2015). Regulation of Nrf2 signaling and longevity in naturally long-lived rodents. Proc. Natl. Acad. Sci. USA.

[22] Inouye S., Hatori Y., Kubo T., Saito S., Kitamura H., Akagi R. (2018). NRF2 and HSF1 coordinately regulate heme oxygenase-1 expression. Biochem. Biophys. Res. Commun..

[23] Zhang M., An C., Gao Y., Leak R.K., Chen J., Zhang F. (2013). Emerging roles of Nrf2 and phase II antioxidant enzymes in neuroprotection. Prog. Neurobiol..

[24] Hou Y., Wang Y., He Q., Li L., Xie H., Zhao Y., Zhao J. (2018). Nrf2 inhibits NLRP3 inflammasome activation through regulating Trx1/TXNIP complex in cerebral ischemia reperfusion injury. Behav. Brain Res..

[25] Kelley N., Jeltema D., Duan Y., He Y. (2019). The NLRP3 Inflammasome: An Overview of Mechanisms of Activation and Regulation. Int. J. Mol. Sci..

[26] Ryter S.W., Ma K.C., Choi A.M.K. (2018). Carbon monoxide in lung cell physiology and disease. Am. J. Physiol. Cell Physiol..

[27] Motterlini R., Otterbein L.E. (2010). The therapeutic potential of carbon monoxide. Nat. Rev. Drug Discov..

[28] Nguyen T., Nioi P., Pickett C.B. (2009). The Nrf2-antioxidant response element signaling pathway and its activation by oxidative stress. J. Biol. Chem..

[29] Villeneuve N.F., Lau A., Zhang D.D. (2010). Regulation of the Nrf2-Keap1 antioxidant response by the ubiquitin proteasome system: An insight into cullin-ring ubiquitin ligases. Antioxid. Redox Signal..

[30] Joshi G., Johnson J.A. (2012). The Nrf2-ARE pathway: A valuable therapeutic target for the treatment of neurodegenerative diseases. Recent Pat. CNS Drug Discov..

[31] McCord J.M., Gao B., Hybertson B.M. (2023). The Complex Genetic and Epigenetic Regulation of the Nrf2 Pathways: A Review. Antioxidants.

[32] Kopacz A., Kloska D., Forman H.J., Jozkowicz A., Grochot-Przeczek A. (2020). Beyond repression of Nrf2: An update on Keap1. Free Radic. Biol. Med..

[33] He F., Ru X., Wen T. (2020). NRF2, a Transcription Factor for Stress Response and Beyond. Int. J. Mol. Sci..

[34] Jaganjac M., Milkovic L., Sunjic S.B., Zarkovic N. (2020). The NRF2, Thioredoxin, and Glutathione System in Tumorigenesis and Anticancer Therapies. Antioxidants.

[35] Sodani K., Patel A., Kathawala R.J., Chen Z.S. (2012). Multidrug resistance associated proteins in multidrug resistance. Chin. J. Cancer.

[36] Ma Q. (2013). Role of nrf2 in oxidative stress and toxicity. Annu. Rev. Pharmacol. Toxicol..

[37] Baird L., Yamamoto M. (2020). The Molecular Mechanisms Regulating the KEAP1-NRF2 Pathway. Mol. Cell. Biol..

[38] Kobayashi M., Yamamoto M. (2006). Nrf2-Keap1 regulation of cellular defense mechanisms against electrophiles and reactive oxygen species. Adv. Enzym. Regul..

[39] Ogura T., Tong K.I., Mio K., Maruyama Y., Kurokawa H., Sato C., Yamamoto M. (2010). Keap1 is a forked-stem dimer structure with two large spheres enclosing the intervening, double glycine repeat, and C-terminal domains. Proc. Natl. Acad. Sci. USA.

[40] Yamamoto T., Suzuki T., Kobayashi A., Wakabayashi J., Maher J., Motohashi H., Yamamoto M. (2008). Physiological significance of reactive cysteine residues of Keap1 in determining Nrf2 activity. Mol. Cell. Biol..

[41] Fukutomi T., Takagi K., Mizushima T., Ohuchi N., Yamamoto M. (2014). Kinetic, thermodynamic, and structural characterizations of the association between Nrf2-DLGex degron and Keap1. Mol. Cell. Biol..

[42] Itoh K., Wakabayashi N., Katoh Y., Ishii T., Igarashi K., Engel J.D., Yamamoto M. (1999). Keap1 represses nuclear activation of antioxidant responsive elements by Nrf2 through binding to the amino-terminal Neh2 domain. Genes. Dev..

[43] Eggler A.L., Liu G., Pezzuto J.M., van Breemen R.B., Mesecar A.D. (2005). Modifying specific cysteines of the electrophile-sensing human Keap1 protein is insufficient to disrupt binding to the Nrf2 domain Neh2. Proc. Natl. Acad. Sci. USA.

[44] Turpaev K.T. (2013). Keap1-Nrf2 signaling pathway: Mechanisms of regulation and role in protection of cells against toxicity caused by xenobiotics and electrophiles. Biochemistry.

[45] Kopacz A., Rojo A.I., Patibandla C., Lastra-Martínez D., Piechota-Polanczyk A., Kloska D., Jozkowicz A., Sutherland C., Cuadrado A., Grochot-Przeczek A. (2022). Overlooked and valuable facts to know in the NRF2/KEAP1 field. Free Radic. Biol. Med..

[46] Baird L., Swift S., Llères D., Dinkova-Kostova A.T. (2014). Monitoring Keap1-Nrf2 interactions in single live cells. Biotechnol. Adv..

[47] Walters T.S., McIntosh D.J., Ingram S.M., Tillery L., Motley E.D., Arinze I.J., Misra S. (2021). SUMO-Modification of Human Nrf2 at K(110) and K(533) Regulates Its Nucleocytoplasmic Localization, Stability and Transcriptional Activity. Cell. Physiol. Biochem..

[48] Katoh Y., Itoh K., Yoshida E., Miyagishi M., Fukamizu A., Yamamoto M. (2001). Two domains of Nrf2 cooperatively bind CBP, a CREB binding protein, and synergistically activate transcription. Genes Cells.

[49] Yang X., Park S.H., Chang H.C., Shapiro J.S., Vassilopoulos A., Sawicki K.T., Chen C., Shang M., Burridge P.W., Epting C.L. (2017). Sirtuin 2 regulates cellular iron homeostasis via deacetylation of transcription factor NRF2. J. Clin. Investig..

[50] Apopa P.L., He X., Ma Q. (2008). Phosphorylation of Nrf2 in the transcription activation domain by casein kinase 2 (CK2) is critical for the nuclear translocation and transcription activation function of Nrf2 in IMR-32 neuroblastoma cells. J. Biochem. Mol. Toxicol..

[51] Sun Z., Huang Z., Zhang D.D. (2009). Phosphorylation of Nrf2 at multiple sites by MAP kinases has a limited contribution in modulating the Nrf2-dependent antioxidant response. PLoS ONE.

[52] Alam J., Igarashi K., Immenschuh S., Shibahara S., Tyrrell R.M. (2004). Regulation of heme oxygenase-1 gene transcription: Recent advances and highlights from the International Conference (Uppsala, 2003) on Heme Oxygenase. Antioxid. Redox Signal..

[53] Reichard J.F., Motz G.T., Puga A. (2007). Heme oxygenase-1 induction by NRF2 requires inactivation of the transcriptional repressor BACH1. Nucleic Acids Res..

[54] Andrés C.M.C., Pérez de la Lastra J.M., Juan C.A., Plou F.J., Pérez-Lebeña E. (2023). Polyphenols as Antioxidant/Pro-Oxidant Compounds and Donors of Reducing Species: Relationship with Human Antioxidant Metabolism. Processes.

[55] Tsuneyoshi T. (2020). BACH1 mediates the antioxidant properties of aged garlic extract. Exp. Ther. Med..

[56] Dhakshinamoorthy S., Jain A.K., Bloom D.A., Jaiswal A.K. (2005). Bach1 competes with Nrf2 leading to negative regulation of the antioxidant response element (ARE)-mediated NAD(P)H:quinone oxidoreductase 1 gene expression and induction in response to antioxidants. J. Biol. Chem..

[57] Fischhuber K., Matzinger M., Heiss E.H. (2020). AMPK Enhances Transcription of Selected Nrf2 Target Genes via Negative Regulation of Bach1. Front. Cell Dev. Biol..

[58] Davies K.J.A., Forman H.J. (2019). Does Bach1 & c-Myc dependent redox dysregulation of Nrf2 & adaptive homeostasis decrease cancer risk in ageing?. Free Radic. Biol. Med..

[59] Li D., Sun D., Zhu Y. (2021). Expression of nuclear factor erythroid-2-related factor 2, broad complex-tramtrack-bric a brac and Cap’n’collar homology 1 and γ-glutamic acid cysteine synthase in peripheral blood of patients with chronic obstructive pulmonary disease and its clinical significance. Exp. Ther. Med..

[60] Liu T., Zhang L., Joo D., Sun S.C. (2017). NF-κB signaling in inflammation. Signal Transduct. Target. Ther..

[61] Krajka-Kuźniak V., Baer-Dubowska W. (2021). Modulation of Nrf2 and NF-κB Signaling Pathways by Naturally Occurring Compounds in Relation to Cancer Prevention and Therapy. Are Combinations Better Than Single Compounds?. Int. J. Mol. Sci..

[62] Allocati N., Masulli M., Di Ilio C., Federici L. (2018). Glutathione transferases: Substrates, inihibitors and pro-drugs in cancer and neurodegenerative diseases. Oncogenesis.

[63] Hine C.M., Mitchell J.R. (2012). NRF2 and the Phase II Response in Acute Stress Resistance Induced by Dietary Restriction. J. Clin. Exp. Pathol..

[64] Rowland A., Miners J.O., Mackenzie P.I. (2013). The UDP-glucuronosyltransferases: Their role in drug metabolism and detoxification. Int. J. Biochem. Cell Biol..

[65] Buckley D.B., Klaassen C.D. (2009). Induction of mouse UDP-glucuronosyltransferase mRNA expression in liver and intestine by activators of aryl-hydrocarbon receptor, constitutive androstane receptor, pregnane X receptor, peroxisome proliferator-activated receptor alpha, and nuclear factor erythroid 2-related factor 2. Drug Metab. Dispos..

[66] Sim E., Abuhammad A., Ryan A. (2014). Arylamine N-acetyltransferases: From drug metabolism and pharmacogenetics to drug discovery. Br. J. Pharmacol..

[67] Pedersen L.C., Yi M., Pedersen L.G., Kaminski A.M. (2022). From Steroid and Drug Metabolism to Glycobiology, Using Sulfotransferase Structures to Understand and Tailor Function. Drug Metab. Dispos..

[68] Gautheron J., Jéru I. (2020). The Multifaceted Role of Epoxide Hydrolases in Human Health and Disease. Int. J. Mol. Sci..

[69] Vilander L.M., Vaara S.T., Donner K.M., Lakkisto P., Kaunisto M.A., Pettilä V. (2019). Heme oxygenase-1 repeat polymorphism in septic acute kidney injury. PLoS ONE.

[70] Ross D., Siegel D. (2021). The diverse functionality of NQO1 and its roles in redox control. Redox Biol..

[71] Lu S.C. (2009). Regulation of glutathione synthesis. Mol. Aspects Med..

[72] Knovich M.A., Storey J.A., Coffman L.G., Torti S.V., Torti F.M. (2009). Ferritin for the clinician. Blood Rev..

[73] Zhang Y., Schwab M. (2011). Phase II Enzymes. Encyclopedia of Cancer.

[74] Childs B.G., Durik M., Baker D.J., van Deursen J.M. (2015). Cellular senescence in aging and age-related disease: From mechanisms to therapy. Nat. Med..

[75] He S., Sharpless N.E. (2017). Senescence in Health and Disease. Cell.

[76] Juan C.A., Pérez de la Lastra J.M., Plou F.J., Pérez-Lebeña E. (2021). The Chemistry of Reactive Oxygen Species (ROS) Revisited: Outlining Their Role in Biological Macromolecules (DNA, Lipids and Proteins) and Induced Pathologies. Int. J. Mol. Sci..

[77] Roger L., Tomas F., Gire V. (2021). Mechanisms and Regulation of Cellular Senescence. Int. J. Mol. Sci..

[78] Victorelli S., Passos J.F. (2017). Telomeres and Cell Senescence–Size Matters Not. EBioMedicine.

[79] Di Micco R., Krizhanovsky V., Baker D., d’Adda di Fagagna F. (2021). Cellular senescence in ageing: From mechanisms to therapeutic opportunities. Nat. Rev. Mol. Cell Biol..

[80] Nousis L., Kanavaros P., Barbouti A. (2023). Oxidative Stress-Induced Cellular Senescence: Is Labile Iron the Connecting Link?. Antioxidants.

[81] Yuan H., Xu Y., Luo Y., Wang N.X., Xiao J.H. (2021). Role of Nrf2 in cell senescence regulation. Mol. Cell Biochem..

[82] Curieses Andrés C.M., Pérez de la Lastra J.M., Andrés Juan C., Plou F.J., Pérez-Lebeña E. (2023). From reactive species to disease development: Effect of oxidants and antioxidants on the cellular biomarkers. J. Biochem. Mol. Toxicol..

[83] Parzych K.R., Klionsky D.J. (2014). An overview of autophagy: Morphology, mechanism, and regulation. Antioxid. Redox Signal..

[84] Bellezza I., Giambanco I., Minelli A., Donato R. (2018). Nrf2-Keap1 signaling in oxidative and reductive stress. Biochim. Biophys. Acta Mol. Cell Res..

[85] Fu W., Liu Y., Yin H. (2019). Mitochondrial Dynamics: Biogenesis, Fission, Fusion, and Mitophagy in the Regulation of Stem Cell Behaviors. Stem Cells Int..

[86] Lastra D., Escoll M., Cuadrado A. (2022). Transcription Factor NRF2 Participates in Cell Cycle Progression at the Level of G1/S and Mitotic Checkpoints. Antioxidants.

[87] Murata H., Takamatsu H., Liu S., Kataoka K., Huh N.H., Sakaguchi M. (2015). NRF2 Regulates PINK1 Expression under Oxidative Stress Conditions. PLoS ONE.

[88] Chan N.C., Salazar A.M., Pham A.H., Sweredoski M.J., Kolawa N.J., Graham R.L., Hess S., Chan D.C. (2011). Broad activation of the ubiquitin-proteasome system by Parkin is critical for mitophagy. Hum. Mol. Genet..

[89] Villavicencio Tejo F., Quintanilla R.A. (2021). Contribution of the Nrf2 Pathway on Oxidative Damage and Mitochondrial Failure in Parkinson and Alzheimer’s Disease. Antioxidants.

[90] Kang L., Liu S., Li J., Tian Y., Xue Y., Liu X. (2020). Parkin and Nrf2 prevent oxidative stress-induced apoptosis in intervertebral endplate chondrocytes via inducing mitophagy and anti-oxidant defenses. Life Sci..

[91] Mi L., Hu J., Li N., Gao J., Huo R., Peng X., Zhang N., Liu Y., Zhao H., Liu R. (2022). The Mechanism of Stem Cell Aging. Stem Cell Rev. Rep..

[92] Liu L., Rando T.A. (2011). Manifestations and mechanisms of stem cell aging. J. Cell Biol..

[93] Schmidlin C.J., Dodson M.B., Madhavan L., Zhang D.D. (2019). Redox regulation by NRF2 in aging and disease. Free Radic. Biol. Med..

[94] Ray S., Corenblum M.J., Anandhan A., Reed A., Ortiz F.O., Zhang D.D., Barnes C.A., Madhavan L. (2018). A Role for Nrf2 Expression in Defining the Aging of Hippocampal Neural Stem Cells. Cell Transplant..

[95] Andrés C.M.C., Pérez de la Lastra J.M., Juan C.A., Plou F.J., Pérez-Lebeña E. (2024). Antioxidant Metabolism Pathways in Vitamins, Polyphenols, and Selenium: Parallels and Divergences. Int. J. Mol. Sci..

[96] Stefanson A.L., Bakovic M. (2014). Dietary regulation of Keap1/Nrf2/ARE pathway: Focus on plant-derived compounds and trace minerals. Nutrients.

[97] Luo Y., Eggler A.L., Liu D., Liu G., Mesecar A.D., van Breemen R.B. (2007). Sites of alkylation of human Keap1 by natural chemoprevention agents. J. Am. Soc. Mass. Spectrom..

[98] Zhang D.D., Hannink M. (2003). Distinct cysteine residues in Keap1 are required for Keap1-dependent ubiquitination of Nrf2 and for stabilization of Nrf2 by chemopreventive agents and oxidative stress. Mol. Cell. Biol..

[99] Ohnuma T., Nakayama S., Anan E., Nishiyama T., Ogura K., Hiratsuka A. (2010). Activation of the Nrf2/ARE pathway via S-alkylation of cysteine 151 in the chemopreventive agent-sensor Keap1 protein by falcarindiol, a conjugated diacetylene compound. Toxicol. Appl. Pharmacol..

[100] Abdel-Massih R.M., Debs E., Othman L., Attieh J., Cabrerizo F.M. (2023). Glucosinolates, a natural chemical arsenal: More to tell than the myrosinase story. Front. Microbiol..

[101] Bhatwalkar S.B., Mondal R., Krishna S.B.N., Adam J.K., Govender P., Anupam R. (2021). Antibacterial Properties of Organosulfur Compounds of Garlic (*Allium sativum*). Front. Microbiol..

[102] Schultz T.W., Yarbrough J.W., Hunter R.S., Aptula A.O. (2007). Verification of the structural alerts for Michael acceptors. Chem. Res. Toxicol..

[103] Little R.D., Masjedizadeh M.R., Wallquist O., Mcloughlin J.I. (2004). The Intramolecular Michael Reaction. Org. React..

[104] Jackson P.A., Widen J.C., Harki D.A., Brummond K.M. (2017). Covalent Modifiers: A Chemical Perspective on the Reactivity of α,β-Unsaturated Carbonyls with Thiols via Hetero-Michael Addition Reactions. J. Med. Chem..

[105] de Freitas Silva M., Pruccoli L., Morroni F., Sita G., Seghetti F., Viegas C., Tarozzi A. (2018). The Keap1/Nrf2-ARE Pathway as a Pharmacological Target for Chalcones. Molecules.

[106] Chu H.W., Sethy B., Hsieh P.W., Horng J.T. (2021). Identification of Potential Drug Targets of Broad-Spectrum Inhibitors with a Michael Acceptor Moiety Using Shotgun Proteomics. Viruses.

[107] Ansari M.I., Khan M.M., Saquib M., Khatoon S., Hussain M.K. (2018). Dithiolethiones: A privileged pharmacophore for anticancer therapy and chemoprevention. Future Med. Chem..

[108] Amalraj A., Pius A., Gopi S., Gopi S. (2017). Biological activities of curcuminoids, other biomolecules from turmeric and their derivatives—A review. J. Tradit. Complement. Med..

[109] Rammohan A., Reddy J.S., Sravya G., Rao C.N., Zyryanov G.V. (2020). Chalcone synthesis, properties and medicinal applications: A review. Environ. Chem. Lett..

[110] Zhuang C., Zhang W., Sheng C., Zhang W., Xing C., Miao Z. (2017). Chalcone: A Privileged Structure in Medicinal Chemistry. Chem. Rev..

[111] Rajendran G., Bhanu D., Aruchamy B., Ramani P., Pandurangan N., Bobba K.N., Oh E.J., Chung H.Y., Gangadaran P., Ahn B.C. (2022). Chalcone: A Promising Bioactive Scaffold in Medicinal Chemistry. Pharmaceuticals.

[112] Bolton J.L., Dunlap T. (2017). Formation and Biological Targets of Quinones: Cytotoxic versus Cytoprotective Effects. Chem. Res. Toxicol..

[113] Linder M.C., Hazegh-Azam M. (1996). Copper biochemistry and molecular biology. Am. J. Clin. Nutr..

[114] Wang X.J., Hayes J.D., Higgins L.G., Wolf C.R., Dinkova-Kostova A.T. (2010). Activation of the NRF2 signaling pathway by copper-mediated redox cycling of para- and ortho-hydroquinones. Chem. Biol..

[115] Satoh T., Saitoh S., Hosaka M., Kosaka K. (2009). Simple ortho- and para-hydroquinones as compounds neuroprotective against oxidative stress in a manner associated with specific transcriptional activation. Biochem. Biophys. Res. Commun..

[116] Bensasson R.V., Zoete V., Dinkova-Kostova A.T., Talalay P. (2008). Two-step mechanism of induction of the gene expression of a prototypic cancer-protective enzyme by diphenols. Chem. Res. Toxicol..

[117] Sharifi-Rad J., Cruz-Martins N., López-Jornet P., Lopez E.P., Harun N., Yeskaliyeva B., Beyatli A., Sytar O., Shaheen S., Sharopov F. (2021). Natural Coumarins: Exploring the Pharmacological Complexity and Underlying Molecular Mechanisms. Oxid. Med. Cell Longev..

[118] Singh B., Sharma R.A. (2015). Plant terpenes: Defense responses, phylogenetic analysis, regulation and clinical applications. 3 Biotech.

[119] Siraj M.A., Islam M.A., Al Fahad M.A., Kheya H.R., Xiao J., Simal-Gandara J. (2021). Cancer Chemopreventive Role of Dietary Terpenoids by Modulating Keap1-Nrf2-ARE Signaling System—A Comprehensive Update. Appl. Sci..

[120] Kalantari K., Moniri M., Boroumand Moghaddam A., Abdul Rahim R., Bin Ariff A., Izadiyan Z., Mohamad R. (2017). A Review of the Biomedical Applications of Zerumbone and the Techniques for Its Extraction from Ginger Rhizomes. Molecules.

[121] Liang S.-T., Chen C., Chen R.-X., Li R., Chen W.-L., Jiang G.-H., Du L.-L. (2022). Michael acceptor molecules in natural products and their mechanism of action. Front. Pharmacol..

[122] Andrés C.M.C., Pérez de la Lastra J.M., Andrés Juan C., Plou F.J., Pérez-Lebeña E. (2023). Chemistry of Hydrogen Sulfide-Pathological and Physiological Functions in Mammalian Cells. Cells.

[123] Munteanu C., Rotariu M., Turnea M., Dogaru G., Popescu C., Spînu A., Andone I., Postoiu R., Ionescu E.V., Oprea C. (2022). Recent Advances in Molecular Research on Hydrogen Sulfide (H(2)S) Role in Diabetes Mellitus (DM)-A Systematic Review. Int. J. Mol. Sci..

[124] Kimura H. (2014). The physiological role of hydrogen sulfide and beyond. Nitric Oxide.

[125] Sen U., Sathnur P.B., Kundu S., Givvimani S., Coley D.M., Mishra P.K., Qipshidze N., Tyagi N., Metreveli N., Tyagi S.C. (2012). Increased endogenous H_2_S generation by CBS, CSE, and 3MST gene therapy improves ex vivo renovascular relaxation in hyperhomocysteinemia. Am. J. Physiol. Cell Physiol..

[126] Dordević D., Jančíková S., Vítězová M., Kushkevych I. (2021). Hydrogen sulfide toxicity in the gut environment: Meta-analysis of sulfate-reducing and lactic acid bacteria in inflammatory processes. J. Adv. Res..

[127] Jandhyala S.M., Talukdar R., Subramanyam C., Vuyyuru H., Sasikala M., Nageshwar Reddy D. (2015). Role of the normal gut microbiota. World J. Gastroenterol..

[128] Abe K., Kimura H. (1996). The possible role of hydrogen sulfide as an endogenous neuromodulator. J. Neurosci..

[129] Hosoki R., Matsuki N., Kimura H. (1997). The possible role of hydrogen sulfide as an endogenous smooth muscle relaxant in synergy with nitric oxide. Biochem. Biophys. Res. Commun..

[130] Shibuya N., Mikami Y., Kimura Y., Nagahara N., Kimura H. (2009). Vascular endothelium expresses 3-mercaptopyruvate sulfurtransferase and produces hydrogen sulfide. J. Biochem..

[131] Wang R. (2012). Physiological implications of hydrogen sulfide: A whiff exploration that blossomed. Physiol. Rev..

[132] Buret A.G., Allain T., Motta J.P., Wallace J.L. (2022). Effects of Hydrogen Sulfide on the Microbiome: From Toxicity to Therapy. Antioxid. Redox Signal..

[133] Kolluru G.K., Shen X., Bir S.C., Kevil C.G. (2013). Hydrogen sulfide chemical biology: Pathophysiological roles and detection. Nitric Oxide.

[134] Xiao Q., Ying J., Xiang L., Zhang C. (2018). The biologic effect of hydrogen sulfide and its function in various diseases. Medicine.

[135] Lobo V., Patil A., Phatak A., Chandra N. (2010). Free radicals, antioxidants and functional foods: Impact on human health. Pharmacogn. Rev..

[136] Olson K.R. (2009). Is hydrogen sulfide a circulating "gasotransmitter" in vertebrate blood?. Biochim. Biophys. Acta.

[137] Kimura H., Shibuya N., Kimura Y. (2012). Hydrogen sulfide is a signaling molecule and a cytoprotectant. Antioxid. Redox Signal..

[138] Ju Y., Fu M., Stokes E., Wu L., Yang G. (2017). H₂S-Mediated Protein S-Sulfhydration: A Prediction for Its Formation and Regulation. Molecules.

[139] Aroca Á., Serna A., Gotor C., Romero L.C. (2015). S-sulfhydration: A cysteine posttranslational modification in plant systems. Plant Physiol..

[140] Andrés C.M.C., Pérez de la Lastra J.M., Andrés Juan C., Plou F.J., Pérez-Lebeña E. (2022). Impact of Reactive Species on Amino Acids;Biological Relevance in Proteins and Induced Pathologies. Int. J. Mol. Sci..

[141] England K., Cotter T.G. (2005). Direct oxidative modifications of signalling proteins in mammalian cells and their effects on apoptosis. Redox Rep..

[142] Yang G., Zhao K., Ju Y., Mani S., Cao Q., Puukila S., Khaper N., Wu L., Wang R. (2013). Hydrogen sulfide protects against cellular senescence via S-sulfhydration of Keap1 and activation of Nrf2. Antioxid. Redox Signal..

[143] Lv B., Chen S., Tang C., Jin H., Du J., Huang Y. (2021). Hydrogen sulfide and vascular regulation*–*An update. J. Adv. Res..

[144] Shen Y., Shen Z., Luo S., Guo W., Zhu Y.Z. (2015). The Cardioprotective Effects of Hydrogen Sulfide in Heart Diseases: From Molecular Mechanisms to Therapeutic Potential. Oxid. Med. Cell Longev..

[145] Pan L.L., Qin M., Liu X.H., Zhu Y.Z. (2017). The Role of Hydrogen Sulfide on Cardiovascular Homeostasis: An Overview with Update on Immunomodulation. Front. Pharmacol..

[146] Kolluru G.K., Shackelford R.E., Shen X., Dominic P., Kevil C.G. (2023). Sulfide regulation of cardiovascular function in health and disease. Nat. Rev. Cardiol..

[147] Meng G., Ma Y., Xie L., Ferro A., Ji Y. (2015). Emerging role of hydrogen sulfide in hypertension and related cardiovascular diseases. Br. J. Pharmacol..

[148] Hu Q., Lukesh J.C. (2023). H(2)S Donors with Cytoprotective Effects in Models of MI/R Injury and Chemotherapy-Induced Cardiotoxicity. Antioxidants.

[149] Deng N.H., Luo W., Gui D.D., Yan B.J., Zhou K., Tian K.J., Ren Z., Xiong W.H., Jiang Z.S. (2022). Hydrogen sulfide plays a potential alternative for the treatment of metabolic disorders of diabetic cardiomyopathy. Mol. Cell. Biochem..

[150] Piragine E., Malanima M.A., Lucenteforte E., Martelli A., Calderone V. (2023). Circulating Levels of Hydrogen Sulfide (H(2)S) in Patients with Age-Related Diseases: A Systematic Review and Meta-Analysis. Biomolecules.

[151] Felipe Salech M., Rafael Jara L., Luis Michea A. (2012). Cambios fisiológicos asociados al envejecimiento. Rev. Médica Clínica Las Condes.

[152] Qabazard B., Stürzenbaum S.R. (2015). H_2_S: A New Approach to Lifespan Enhancement and Healthy Ageing?. Handb. Exp. Pharmacol..

[153] Huerta de la Cruz S., Rodríguez-Palma E.J., Santiago-Castañeda C.L., Beltrán-Ornelas J.H., Sánchez-López A., Rocha L., Centurión D. (2022). Exogenous hydrogen sulfide restores CSE and CBS but no 3-MST protein expression in the hypothalamus and brainstem after severe traumatic brain injury. Metab. Brain Dis..

[154] Münke M., Kraus J.P., Ohura T., Francke U. (1988). The gene for cystathionine beta-synthase (CBS) maps to the subtelomeric region on human chromosome 21q and to proximal mouse chromosome 17. Am. J. Hum. Genet..

[155] Yin P., Zhao C., Li Z., Mei C., Yao W., Liu Y., Li N., Qi J., Wang L., Shi Y. (2012). Sp1 is involved in regulation of cystathionine γ-lyase gene expression and biological function by PI3K/Akt pathway in human hepatocellular carcinoma cell lines. Cell. Signal..

[156] Rodrigues C., Percival S.S. (2019). Immunomodulatory Effects of Glutathione, Garlic Derivatives, and Hydrogen Sulfide. Nutrients.

[157] Shang A., Cao S.Y., Xu X.Y., Gan R.Y., Tang G.Y., Corke H., Mavumengwana V., Li H.B. (2019). Bioactive Compounds and Biological Functions of Garlic (*Allium sativum* L.). Foods.

[158] Najman K., Sadowska A., Hallmann E. (2021). Evaluation of Bioactive and Physicochemical Properties of White and Black Garlic (*Allium sativum* L.) from Conventional and Organic Cultivation. Appl. Sci..

[159] Fimognari C., Turrini E., Ferruzzi L., Lenzi M., Hrelia P. (2012). Natural isothiocyanates: Genotoxic potential versus chemoprevention. Mutat. Res..

[160] Zhang Y. (2012). The 1,2-benzenedithiole-based cyclocondensation assay: A valuable tool for the measurement of chemopreventive isothiocyanates. Crit. Rev. Food Sci. Nutr..

[161] Li Z., Liu Y., Fang Z., Yang L., Zhuang M., Zhang Y., Zhao W., Sun P. (2014). Variation of Sulforaphane Levels in Broccoli (*Brassica oleracea* var. *Italica*) during Flower Development and the Role of Gene AOP2. J. Liq. Chromatogr. Relat. Technol..

[162] Chiu Y.C., Matak K., Ku K.M. (2019). Methyl jasmonate treated broccoli: Impact on the production of glucosinolates and consumer preferences. Food Chem..

[163] Yadav K., Dhankhar J. (2022). Isothiocyanates—A Review of their Health Benefits and Potential Food Applications. Curr. Res. Nutr. Food Sci..

[164] Maruthupandy M., Seo J. (2019). Allyl isothiocyanate encapsulated halloysite covered with polyacrylate as a potential antibacterial agent against food spoilage bacteria. Mater. Sci. Eng. C Mater. Biol. Appl..

[165] Castellano R., Perruchot M.H., Tesseraud S., Métayer-Coustard S., Baeza E., Mercier Y., Gondret F. (2017). Methionine and cysteine deficiencies altered proliferation rate and time-course differentiation of porcine preadipose cells. Amino Acids.

[166] Krishnaswamy K., Rao S.B. (1977). Failure to produce atherosclerosis in Macaca radiata on a high-methionine, high-fat, pyridoxine-deficient diet. Atherosclerosis.

[167] Mikkelsen M.D., Petersen B., Olsen C., Halkier B. (2002). Biosynthesis and metabolic engineering of glucosinolates. Amino Acids.

[168] Chan Y.A., Podevels A.M., Kevany B.M., Thomas M.G. (2009). Biosynthesis of polyketide synthase extender units. Nat. Prod. Rep..

[169] Kachungwa Lugata J., Ortega A.D.S.V., Szabó C. (2022). The Role of Methionine Supplementation on Oxidative Stress and Antioxidant Status of Poultry-A Review. Agriculture.

[170] Liu X.Y., Li C.Y., Bu H., Li Z., Li B., Sun M.M., Guo Y.S., Zhang L., Ren W.B., Fan Z.L. (2008). The neuroprotective potential of phase II enzyme inducer on motor neuron survival in traumatic spinal cord injury in vitro. Cell. Mol. Neurobiol..

[171] Brown D.A., Betharia S., Yen J.H., Tran Q., Mistry H., Smith K. (2014). Synthesis and structure-activity relationships study of dithiolethiones as inducers of glutathione in the SH-SY5Y neuroblastoma cell line. Bioorg. Med. Chem. Lett..

[172] Begleiter A., Leith M.K., Curphey T.J., Doherty G.P. (1997). Induction of DT-diaphorase in cancer chemoprevention and chemotherapy. Oncol. Res..

[173] Zipper L.M., Mulcahy R.T. (2002). The Keap1 BTB/POZ dimerization function is required to sequester Nrf2 in cytoplasm. J. Biol. Chem..

[174] Sekhar K.R., Spitz D.R., Harris S., Nguyen T.T., Meredith M.J., Holt J.T., Gius D., Marnett L.J., Summar M.L., Freeman M.L. (2002). Redox-sensitive interaction between KIAA0132 and Nrf2 mediates indomethacin-induced expression of gamma-glutamylcysteine synthetase. Free Radic. Biol. Med..

[175] Li K.R., Yang S.Q., Gong Y.Q., Yang H., Li X.M., Zhao Y.X., Yao J., Jiang Q., Cao C. (2016). 3H-1,2-dithiole-3-thione protects retinal pigment epithelium cells against Ultra-violet radiation via activation of Akt-mTORC1-dependent Nrf2-HO-1 signaling. Sci. Rep..

[176] Kuo P.C., Brown D.A., Scofield B.A., Yu I.C., Chang F.L., Wang P.Y., Yen J.H. (2016). 3H-1,2-dithiole-3-thione as a novel therapeutic agent for the treatment of experimental autoimmune encephalomyelitis. Brain Behav. Immun..

[177] Karuri A.R., Huang Y., Bodreddigari S., Sutter C.H., Roebuck B.D., Kensler T.W., Sutter T.R. (2006). 3H-1,2-dithiole-3-thione targets nuclear factor kappaB to block expression of inducible nitric-oxide synthase, prevents hypotension, and improves survival in endotoxemic rats. J. Pharmacol. Exp. Ther..

[178] Kuo P.-C., Yu I.C., Scofield B.A., Brown D.A., Curfman E.T., Paraiso H.C., Chang F.-L., Yen J.-H. (2017). 3H-1,2-Dithiole-3-thione as a novel therapeutic agent for the treatment of ischemic stroke through Nrf2 defense pathway. Brain Behav. Immun..

[179] Rakitin O.A. (2021). Synthesis and Reactivity of 3H-1,2-dithiole-3-thiones. Molecules.

[180] Huang A., Seité S., Adar T. (2018). The use of balneotherapy in dermatology. Clin. Dermatol..

[181] Nasermoaddeli A., Kagamimori S. (2005). Balneotherapy in medicine: A review. Environ. Health Prev. Med..

[182] Maraver F., Armijo F., Fernandez-Toran M.A., Armijo O., Ejeda J.M., Vazquez I., Corvillo I., Torres-Piles S. (2021). Peloids as Thermotherapeutic Agents. Int. J. Environ. Res. Public Health.

[183] Gálvez I., Torres-Piles S., Ortega-Rincón E. (2018). Balneotherapy, Immune System, and Stress Response: A Hormetic Strategy?. Int. J. Mol. Sci..

[184] Kovács C., Pecze M., Tihanyi Á., Kovács L., Balogh S., Bender T. (2012). The effect of sulphurous water in patients with osteoarthritis of hand. Double-blind, randomized, controlled follow-up study. Clin. Rheumatol..

[185] Vaamonde-García C., Vela-Anero Á., Hermida-Gómez T., Fernández-Burguera E., Filgueira-Fernández P., Goyanes N., Blanco F.J., Meijide-Faílde R. (2020). Effect of balneotherapy in sulfurous water on an in vivo murine model of osteoarthritis. Int. J. Biometeorol..

[186] Protano C., Fontana M., De Giorgi A., Marotta D., Cocomello N., Crucianelli S., Del Cimmuto A., Vitali M. (2023). Balneotherapy for osteoarthritis: A systematic review. Rheumatol. Int..

[187] Brglez Mojzer E., Knez Hrnčič M., Škerget M., Knez Ž., Bren U. (2016). Polyphenols: Extraction Methods, Antioxidative Action, Bioavailability and Anticarcinogenic Effects. Molecules.

[188] Rahman M.M., Rahaman M.S., Islam M.R., Rahman F., Mithi F.M., Alqahtani T., Almikhlafi M.A., Alghamdi S.Q., Alruwaili A.S., Hossain M.S. (2022). Role of Phenolic Compounds in Human Disease: Current Knowledge and Future Prospects. Molecules.

[189] Andrés C.M.C., Pérez de la Lastra J.M., Andrés Juan C., Plou F.J., Pérez-Lebeña E. (2023). Superoxide Anion Chemistry;Its Role at the Core of the Innate Immunity. Int. J. Mol. Sci..

[190] Hamad H.A.M., Farid A.B. (2021). Phenolic Compounds: Classification, Chemistry, and Updated Techniques of Analysis and Synthesis. Phenolic Compounds.

[191] Costa M., Sezgin-Bayindir Z., Losada-Barreiro S., Paiva-Martins F., Saso L., Bravo-Díaz C. (2021). Polyphenols as Antioxidants for Extending Food Shelf-Life and in the Prevention of Health Diseases: Encapsulation and Interfacial Phenomena. Biomedicines.

[192] Amankulova D., Berganayeva G., Kudaibergenova B., Zhetpisbay D., Sharipova A., Dyusebaeva M. (2023). Recent Advances in the Synthesis and Applications of m-Aryloxy Phenols. Molecules.

[193] Qiu Z., Li C.-J. (2020). Transformations of less-activated phenols and phenol derivatives via C–O cleavage. Chem. Rev..

[194] Floris B., Galloni P., Conte V., Sabuzi F. (2021). Tailored Functionalization of Natural Phenols to Improve Biological Activity. Biomolecules.

[195] Tang J., Zhang S., Zhou B.-W., Wang W., Zhao L. (2023). Hyperconjugative Aromaticity-Based Circularly Polarized Luminescence Enhancement in Polyaurated Heterocycles. J. Am. Chem. Soc..

[196] Taranto F., Pasqualone A., Mangini G., Tripodi P., Miazzi M.M., Pavan S., Montemurro C. (2017). Polyphenol Oxidases in Crops: Biochemical, Physiological and Genetic Aspects. Int. J. Mol. Sci..

[197] Liang Y., Were L. (2020). Cysteine’s effects on chlorogenic acid quinone induced greening and browning: Mechanism and effect on antioxidant reducing capacity. Food Chem..

[198] Araji S., Grammer T.A., Gertzen R., Anderson S.D., Mikulic-Petkovsek M., Veberic R., Phu M.L., Solar A., Leslie C.A., Dandekar A.M. (2014). Novel Roles for the Polyphenol Oxidase Enzyme in Secondary Metabolism and the Regulation of Cell Death in Walnut. Plant Physiol..

[199] Bolton J.L., Trush M.A., Penning T.M., Dryhurst G., Monks T.J. (2000). Role of Quinones in Toxicology. Chem. Res. Toxicol..

[200] Unoki T., Akiyama M., Kumagai Y. (2020). Nrf2 Activation and Its Coordination with the Protective Defense Systems in Response to Electrophilic Stress. Int. J. Mol. Sci..

[201] Nogales-Delgado S. (2021). Polyphenoloxidase (PPO): Effect, Current Determination and Inhibition Treatments in Fresh-Cut Produce. Appl. Sci..

[202] Dulo B., Phan K., Githaiga J., Raes K., De Meester S. (2021). Natural Quinone Dyes: A Review on Structure, Extraction Techniques, Analysis and Application Potential. Waste Biomass Valorization.

[203] Varela-López A., Giampieri F., Battino M., Quiles J.L. (2016). Coenzyme Q and Its Role in the Dietary Therapy against Aging. Molecules.

[204] Enriquez J.A., Lenaz G. (2014). Coenzyme q and the respiratory chain: Coenzyme q pool and mitochondrial supercomplexes. Mol. Syndromol..

[205] Nardini M. (2022). Phenolic Compounds in Food: Characterization and Health Benefits. Molecules.

[206] Soliman I.A., Hasanien Y.A., Zaki A.G., Shawky H.A., Nassrallah A.A. (2022). Irradiation impact on biological activities of Anthraquinone pigment produced from Talaromyces purpureogenus and its evaluation, characterization and application in beef burger as natural preservative. BMC Microbiol..

[207] Malik E.M., Müller C.E. (2016). Anthraquinones As Pharmacological Tools and Drugs. Med. Res. Rev..

[208] Siva R., Mayes S., Behera S.K., Rajasekaran C. (2012). Anthraquinones dye production using root cultures of *Oldenlandia umbellata* L. Ind. Crops Prod..

[209] Hart P.W., Rudie A.W. (2014). Anthraquinone a review of the rise and fall of a pulping catalyst. Tappi J..

[210] Goor G., Glenneberg J., Jacobi S. (2007). Hydrogen Peroxide. Ullmann’s Encyclopedia of Industrial Chemistry.

[211] Avery M.L., Humphrey J.S., Primus T.M., Decker D.G., McGrane A.P. (1998). Anthraquinone protects rice seed from birds. Crop Prot..

[212] Wu M., Jing Y., Wong A.A., Fell E.M., Jin S., Tang Z., Gordon R.G., Aziz M.J. (2020). Extremely Stable Anthraquinone Negolytes Synthesized from Common Precursors. Chem.

[213] Locatelli M., Epifano F., Genovese S., Carlucci G., Koncić M.Z., Kosalec I., Kremer D. (2011). Anthraquinone profile, antioxidant and antimicrobial properties of bark extracts of Rhamnus catharticus and R. orbiculatus. Nat. Prod. Commun..

[214] Hu B., Zhang H., Meng X., Wang F., Wang P. (2014). Aloe-emodin from rhubarb (*Rheum rhabarbarum*) inhibits lipopolysaccharide-induced inflammatory responses in RAW264.7 macrophages. J. Ethnopharmacol..

[215] Sanders B., Ray A.M., Goldberg S., Clark T., McDaniel H.R., Atlas S.E., Farooqi A., Konefal J., Lages L.C., Lopez J. (2018). Anti-cancer effects of aloe-emodin: A systematic review. J. Clin. Transl. Res..

[216] Sun W., Wang Z., Sun M., Huang W., Wang Y., Wang Y. (2021). Aloin antagonizes stimulated ischemia/reperfusion-induced damage and inflammatory response in cardiomyocytes by activating the Nrf2/HO-1 defense pathway. Cell Tissue Res..

[217] Paudel P., Jung H.A., Choi J.S. (2018). Anthraquinone and naphthopyrone glycosides from Cassia obtusifolia seeds mediate hepatoprotection via Nrf2-mediated HO-1 activation and MAPK modulation. Arch. Pharm. Res..

[218] Su C., Liu Z., Wang Y., Wang Y., Song E., Song Y. (2017). The electrophilic character of quinones is essential for the suppression of Bach1. Toxicology.

[219] Gaonkar S.L., Vignesh U.N. (2017). Synthesis and pharmacological properties of chalcones: A review. Res. Chem. Intermed..

[220] Karthikeyan C., Moorthy N.S., Ramasamy S., Vanam U., Manivannan E., Karunagaran D., Trivedi P. (2015). Advances in chalcones with anticancer activities. Recent Pat. Anticancer Drug Discov..

[221] Singh P., Anand A., Kumar V. (2014). Recent developments in biological activities of chalcones: A mini review. Eur. J. Med. Chem..

[222] Sahu N.K., Balbhadra S.S., Choudhary J., Kohli D.V. (2012). Exploring pharmacological significance of chalcone scaffold: A review. Curr. Med. Chem..

[223] Batovska D.I., Todorova I.T. (2010). Trends in utilization of the pharmacological potential of chalcones. Curr. Clin. Pharmacol..

[224] López S.N., Castelli M.V., Zacchino S.A., Domínguez J.N., Lobo G., Charris-Charris J., Cortés J.C., Ribas J.C., Devia C., Rodríguez A.M. (2001). In vitro antifungal evaluation and structure-activity relationships of a new series of chalcone derivatives and synthetic analogues, with inhibitory properties against polymers of the fungal cell wall. Bioorg. Med. Chem..

[225] Rastelli G., Antolini L., Benvenuti S., Costantino L. (2000). Structural bases for the inhibition of aldose reductase by phenolic compounds. Bioorg. Med. Chem..

[226] Zhang X.-J., Li L.-Y., Wang S.-S., Que S., Yang W.-Z., Zhang F.-Y., Gong N.-B., Cheng W., Liang H., Ye M. (2013). Oxyfadichalcones A–C: Three chalcone dimers fused through a cyclobutane ring from Tibetan medicine Oxytropis falcata Bunge. Tetrahedron.

[227] Raj L., Ide T., Gurkar A.U., Foley M., Schenone M., Li X., Tolliday N.J., Golub T.R., Carr S.A., Shamji A.F. (2011). Selective killing of cancer cells by a small molecule targeting the stress response to ROS. Nature.

[228] Wang Y., Curtis-Long M.J., Lee B.W., Yuk H.J., Kim D.W., Tan X.F., Park K.H. (2014). Inhibition of tyrosinase activity by polyphenol compounds from Flemingia philippinensis roots. Bioorg. Med. Chem..

[229] Mdee L.K., Yeboah S.O., Abegaz B.M. (2003). Rhuschalcones II-VI, five new bichalcones from the root bark of Rhus pyroides. J. Nat. Prod..

[230] Pereira R., Silva A.M.S., Ribeiro D., Silva V.L.M., Fernandes E. (2023). Bis-chalcones: A review of synthetic methodologies and anti-inflammatory effects. Eur. J. Med. Chem..

[231] Ni L., Meng C.Q., Sikorski J.A. (2004). Recent advances in therapeutic chalcones. Expert Opin. Ther. Pat..

[232] Zhou B., Xing C. (2015). Diverse Molecular Targets for Chalcones with Varied Bioactivities. Med. Chem..

[233] Egbujor M.C., Buttari B., Profumo E., Telkoparan-Akillilar P., Saso L. (2022). An Overview of NRF2-Activating Compounds Bearing α,β-Unsaturated Moiety and Their Antioxidant Effects. Int. J. Mol. Sci..

[234] Sharifi-Rad J., Rayess Y.E., Rizk A.A., Sadaka C., Zgheib R., Zam W., Sestito S., Rapposelli S., Neffe-Skocińska K., Zielińska D. (2020). Turmeric and Its Major Compound Curcumin on Health: Bioactive Effects and Safety Profiles for Food, Pharmaceutical, Biotechnological and Medicinal Applications. Front. Pharmacol..

[235] Fadus M.C., Lau C., Bikhchandani J., Lynch H.T. (2017). Curcumin: An age-old anti-inflammatory and anti-neoplastic agent. J. Tradit. Complement. Med..

[236] Benameur T., Soleti R., Panaro M.A., La Torre M.E., Monda V., Messina G., Porro C. (2021). Curcumin as Prospective Anti-Aging Natural Compound: Focus on Brain. Molecules.

[237] Nebrisi E.E. (2021). Neuroprotective Activities of Curcumin in Parkinson’s Disease: A Review of the Literature. Int. J. Mol. Sci..

[238] Peng Y., Ao M., Dong B., Jiang Y., Yu L., Chen Z., Hu C., Xu R. (2021). Anti-Inflammatory Effects of Curcumin in the Inflammatory Diseases: Status, Limitations and Countermeasures. Drug Des. Devel Ther..

[239] Mansouri K., Rasoulpoor S., Daneshkhah A., Abolfathi S., Salari N., Mohammadi M., Rasoulpoor S., Shabani S. (2020). Clinical effects of curcumin in enhancing cancer therapy: A systematic review. BMC Cancer.

[240] Zoi V., Galani V., Tsekeris P., Kyritsis A.P., Alexiou G.A. (2022). Radiosensitization and Radioprotection by Curcumin in Glioblastoma and Other Cancers. Biomedicines.

[241] Kou H., Huang L., Jin M., He Q., Zhang R., Ma J. (2023). Effect of curcumin on rheumatoid arthritis: A systematic review and meta-analysis. Front. Immunol..

[242] Shin J.W., Chun K.S., Kim D.H., Kim S.J., Kim S.H., Cho N.C., Na H.K., Surh Y.J. (2020). Curcumin induces stabilization of Nrf2 protein through Keap1 cysteine modification. Biochem. Pharmacol..

[243] Cao D., Liu Z., Verwilst P., Koo S., Jangjili P., Kim J.S., Lin W. (2019). Coumarin-Based Small-Molecule Fluorescent Chemosensors. Chem. Rev..

[244] Küpeli Akkol E., Genç Y., Karpuz B., Sobarzo-Sánchez E., Capasso R. (2020). Coumarins and Coumarin-Related Compounds in Pharmacotherapy of Cancer. Cancers.

[245] Dennis R.A.M., Priscilla F., Jennifer P., Meryll D., Sanjeet K. (2022). Phenolic Compounds and Antioxidant Activities of Eight Species of Fabaceae That Are Commonly Used in Traditional Medical Practices in the Republic of Suriname. Medicinal Plants.

[246] Al-Khayri J.M., Rashmi R., Toppo V., Chole P.B., Banadka A., Sudheer W.N., Nagella P., Shehata W.F., Al-Mssallem M.Q., Alessa F.M. (2023). Plant Secondary Metabolites: The Weapons for Biotic Stress Management. Metabolites.

[247] Hassanein E.H.M., Sayed A.M., Hussein O.E., Mahmoud A.M. (2020). Coumarins as Modulators of the Keap1/Nrf2/ARE Signaling Pathway. Oxid. Med. Cell Longev..

[248] Al-Warhi T., Sabt A., Elkaeed E.B., Eldehna W.M. (2020). Recent advancements of coumarin-based anticancer agents: An up-to-date review. Bioorg. Chem..

[249] Garg S.S., Gupta J., Sharma S., Sahu D. (2020). An insight into the therapeutic applications of coumarin compounds and their mechanisms of action. Eur. J. Pharm. Sci..

[250] Jameel E., Umar T., Kumar J., Hoda N. (2016). Coumarin: A Privileged Scaffold for the Design and Development of Antineurodegenerative Agents. Chem. Biol. Drug Des..

[251] Supuran C.T. (2020). Coumarin carbonic anhydrase inhibitors from natural sources. J. Enzym. Inhib. Med. Chem..

[252] Carneiro A., Matos M.J., Uriarte E., Santana L. (2021). Trending Topics on Coumarin and Its Derivatives in 2020. Molecules.

[253] Zhang L., Xu Z. (2019). Coumarin-containing hybrids and their anticancer activities. Eur. J. Med. Chem..

[254] Reddy D.S., Kongot M., Kumar A. (2021). Coumarin hybrid derivatives as promising leads to treat tuberculosis: Recent developments and critical aspects of structural design to exhibit anti-tubercular activity. Tuberculosis.

[255] Xu Z., Chen Q., Zhang Y., Liang C. (2021). Coumarin-based derivatives with potential anti-HIV activity. Fitoterapia.

[256] Feng D., Zhang A., Yang Y., Yang P. (2020). Coumarin-containing hybrids and their antibacterial activities. Arch. Pharm..

[257] Song X.F., Fan J., Liu L., Liu X.F., Gao F. (2020). Coumarin derivatives with anticancer activities: An update. Arch. Pharm..

[258] Annunziata F., Pinna C., Dallavalle S., Tamborini L., Pinto A. (2020). An Overview of Coumarin as a Versatile and Readily Accessible Scaffold with Broad-Ranging Biological Activities. Int. J. Mol. Sci..

[259] Harborne J.B. (1982). The Natural Coumarins: Occurrence, Chemistry and Biochemistry (Book). Plant Cell Environ..

[260] Di Stasi L.C. (2023). Natural Coumarin Derivatives Activating Nrf2 Signaling Pathway as Lead Compounds for the Design and Synthesis of Intestinal Anti-Inflammatory Drugs. Pharmaceuticals.

[261] Dinkova-Kostova A.T., Liby K.T., Stephenson K.K., Holtzclaw W.D., Gao X., Suh N., Williams C., Risingsong R., Honda T., Gribble G.W. (2005). Extremely potent triterpenoid inducers of the phase 2 response: Correlations of protection against oxidant and inflammatory stress. Proc. Natl. Acad. Sci. USA.

[262] Djedjibegovic J., Marjanovic A., Panieri E., Saso L. (2020). Ellagic Acid-Derived Urolithins as Modulators of Oxidative Stress. Oxid. Med. Cell Longev..

[263] Bae G.S., Kim D.G., Jo I.J., Choi S.B., Kim M.J., Shin J.Y., Kim D.U., Song H.J., Joo M., Park S.J. (2019). Heme oxygenase-1 induced by desoxo-narchinol-A attenuated the severity of acute pancreatitis via blockade of neutrophil infiltration. Int. Immunopharmacol..

[264] Kim K.W., Yoon C.S., Kim Y.C., Oh H. (2019). Desoxo-narchinol A and Narchinol B Isolated from Nardostachys jatamansi Exert Anti-neuroinflammatory Effects by Up-regulating of Nuclear Transcription Factor Erythroid-2-Related Factor 2/Heme Oxygenase-1 Signaling. Neurotox. Res..

[265] Tsoyi K., Jang H.J., Lee Y.S., Kim Y.M., Kim H.J., Seo H.G., Lee J.H., Kwak J.H., Lee D.U., Chang K.C. (2011). (+)-Nootkatone and (+)-valencene from rhizomes of Cyperus rotundus increase survival rates in septic mice due to heme oxygenase-1 induction. J. Ethnopharmacol..

[266] Kim N., Hwangbo C., Lee S., Lee J.H. (2013). Eupatolide inhibits PDGF-induced proliferation and migration of aortic smooth muscle cells through ROS-dependent heme oxygenase-1 induction. Phytother. Res..

[267] Prasannan R., Kalesh K.A., Shanmugam M.K., Nachiyappan A., Ramachandran L., Nguyen A.H., Kumar A.P., Lakshmanan M., Ahn K.S., Sethi G. (2012). Key cell signaling pathways modulated by zerumbone: Role in the prevention and treatment of cancer. Biochem. Pharmacol..

[268] Leung W.S., Yang M.L., Lee S.S., Kuo C.W., Ho Y.C., Huang-Liu R., Lin H.W., Kuan Y.H. (2017). Protective effect of zerumbone reduces lipopolysaccharide-induced acute lung injury via antioxidative enzymes and Nrf2/HO-1 pathway. Int. Immunopharmacol..

[269] Ha D.T., Phuong T.T., Oh J., Bae K., Thuan N.D., Na M. (2014). Palbinone from Paeonia suffruticosa protects hepatic cells via up-regulation of heme oxygenase-1. Phytother. Res..

[270] Zhang Y., Liu J., Jia W., Zhao A., Li T. (2005). Distinct immunosuppressive effect by Isodon serra extracts. Int. Immunopharmacol..

[271] Mishra S.K., Sangwan N.S., Sangwan R.S. (2007). Phcog rev.: Plant review Andrographis paniculata (Kalmegh): A review. Pharmacogn. Rev..

[272] Kim Y.M., Kim H.J., Chang K.C. (2015). Glycyrrhizin reduces HMGB1 secretion in lipopolysaccharide-activated RAW 264.7 cells and endotoxemic mice by p38/Nrf2-dependent induction of HO-1. Int. Immunopharmacol..

[273] Mou K., Pan W., Han D., Wen X., Cao F., Miao Y., Li P. (2019). Glycyrrhizin protects human melanocytes from H2O2-induced oxidative damage via the Nrf2-dependent induction of HO-1. Int. J. Mol. Med..

[274] Heyninck K., Sabbe L., Chirumamilla C.S., Szarc Vel Szic K., Vander Veken P., Lemmens K.J.A., Lahtela-Kakkonen M., Naulaerts S., Op de Beeck K., Laukens K. (2016). Withaferin A induces heme oxygenase (HO-1) expression in endothelial cells via activation of the Keap1/Nrf2 pathway. Biochem. Pharmacol..

[275] Chew L.Y., Zhang H., He J., Yu F. (2021). The Nrf2-Keap1 pathway is activated by steroid hormone signaling to govern neuronal remodeling. Cell Rep..

[276] Freeman B.A., Baker P.R., Schopfer F.J., Woodcock S.R., Napolitano A., d’Ischia M. (2008). Nitro-fatty acid formation and signaling. J. Biol. Chem..

[277] Piesche M., Roos J., Kühn B., Fettel J., Hellmuth N., Brat C., Maucher I.V., Awad O., Matrone C., Comerma Steffensen S.G. (2020). The Emerging Therapeutic Potential of Nitro Fatty Acids and Other Michael Acceptor-Containing Drugs for the Treatment of Inflammation and Cancer. Front. Pharmacol..

[278] Kansanen E., Jyrkkänen H.-K., Volger O.L., Leinonen H., Kivelä A.M., Häkkinen S.-K., Woodcock S.R., Schopfer F.J., Horrevoets A.J., Ylä-Herttuala S. (2009). Nrf2-dependent and-independent responses to nitro-fatty acids in human endothelial cells. J. Biol. Chem..

[279] Schopfer F.J., Vitturi D.A., Jorkasky D.K., Freeman B.A. (2018). Nitro-fatty acids: New drug candidates for chronic inflammatory and fibrotic diseases. Nitric Oxide.

[280] Rao P.V., Gan S.H. (2014). Cinnamon: A multifaceted medicinal plant. Evid. Based Complement. Altern. Med..

[281] Huang T.C., Chung Y.L., Wu M.L., Chuang S.M. (2011). Cinnamaldehyde enhances Nrf2 nuclear translocation to upregulate phase II detoxifying enzyme expression in HepG2 cells. J. Agric. Food Chem..

[282] O’Brien J., Wendell S.G. (2020). Electrophile Modulation of Inflammation: A Two-Hit Approach. Metabolites.

[283] Gai C., Harnor S.J., Zhang S., Cano C., Zhuang C., Zhao Q. (2022). Advanced approaches of developing targeted covalent drugs. RSC Med. Chem..

[284] Mons E., Jansen I.D.C., Loboda J., van Doodewaerd B.R., Hermans J., Verdoes M., van Boeckel C.A.A., van Veelen P.A., Turk B., Turk D. (2019). The Alkyne Moiety as a Latent Electrophile in Irreversible Covalent Small Molecule Inhibitors of Cathepsin K. J. Am. Chem. Soc..

[285] Ghosh A.K., Samanta I., Mondal A., Liu W.R. (2019). Covalent Inhibition in Drug Discovery. ChemMedChem.

[286] Roth G.J., Stanford N., Majerus P.W. (1975). Acetylation of prostaglandin synthase by aspirin. Proc. Natl. Acad. Sci. USA.

[287] Boike L., Henning N.J., Nomura D.K. (2022). Advances in covalent drug discovery. Nat. Rev. Drug Discov..

[288] Bauer R.A. (2015). Covalent inhibitors in drug discovery: From accidental discoveries to avoided liabilities and designed therapies. Drug Discov. Today.

[289] Selvaraju K., Mofers A., Pellegrini P., Salomonsson J., Ahlner A., Morad V., Hillert E.K., Espinosa B., Arnér E.S.J., Jensen L. (2019). Cytotoxic unsaturated electrophilic compounds commonly target the ubiquitin proteasome system. Sci. Rep..

[290] Reddi R.N., Rogel A., Gabizon R., Rawale D.G., Harish B., Marom S., Tivon B., Arbel Y.S., Gurwicz N., Oren R. (2023). Sulfamate Acetamides as Self-Immolative Electrophiles for Covalent Ligand-Directed Release Chemistry. J. Am. Chem. Soc..

[291] LoPachin R.M., Gavin T. (2016). Reactions of electrophiles with nucleophilic thiolate sites: Relevance to pathophysiological mechanisms and remediation. Free Radic. Res..

[292] Baron G., Altomare A., Della Vedova L., Gado F., Quagliano O., Casati S., Tosi N., Bresciani L., Del Rio D., Roda G. (2024). Unraveling the parahormetic mechanism underlying the health-protecting effects of grapeseed procyanidins. Redox Biol..

[293] Wang R.C., Wang Z. (2023). Precision Medicine: Disease Subtyping and Tailored Treatment. Cancers.

[294] Satoh T., McKercher S.R., Lipton S.A. (2013). Nrf2/ARE-mediated antioxidant actions of pro-electrophilic drugs. Free Radic. Biol. Med..

[295] Lv Y., Li W., Liao W., Jiang H., Liu Y., Cao J., Lu W., Feng Y. (2024). Nano-Drug Delivery Systems Based on Natural Products. Int. J. Nanomed..

[296] Repash E.M., Pensabene K.M., Palenchar P.M., Eggler A.L. (2021). Solving the Problem of Assessing Synergy and Antagonism for Non-Traditional Dosing Curve Compounds Using the DE/ZI Method: Application to Nrf2 Activators. Front. Pharmacol..

[297] Yang S., Kar S. (2023). Application of artificial intelligence and machine learning in early detection of adverse drug reactions (ADRs) and drug-induced toxicity. Artif. Intell. Chem..

[298] Singh S., Nagalakshmi D., Sharma K.K., Ravichandiran V. (2021). Natural antioxidants for neuroinflammatory disorders and possible involvement of Nrf2 pathway: A review. Heliyon.

[299] Lillich F.F., Imig J.D., Proschak E. (2020). Multi-Target Approaches in Metabolic Syndrome. Front. Pharmacol..

[300] Lavezzi A.M., Ramos-Molina B. (2023). Environmental Exposure Science and Human Health. Int. J. Environ. Res. Public. Health.

[301] Stielow M., Witczyńska A., Kubryń N., Fijałkowski Ł., Nowaczyk J., Nowaczyk A. (2023). The Bioavailability of Drugs-The Current State of Knowledge. Molecules.

[302] Bertelli A., Biagi M., Corsini M., Baini G., Cappellucci G., Miraldi E. (2021). Polyphenols: From Theory to Practice. Foods.

[303] Żyżelewicz D., Oracz J. (2022). Bioavailability and Bioactivity of Plant Antioxidants. Antioxidants.

[304] Di Lorenzo C., Colombo F., Biella S., Stockley C., Restani P. (2021). Polyphenols and Human Health: The Role of Bioavailability. Nutrients.

[305] Almazroo O.A., Miah M.K., Venkataramanan R. (2017). Drug Metabolism in the Liver. Clin. Liver Dis..

[306] Zhang F., He F., Li L., Guo L., Zhang B., Yu S., Zhao W. (2020). Bioavailability Based on the Gut Microbiota: A New Perspective. Microbiol. Mol. Biol. Rev..

[307] Orlando P., Nartea A., Silvestri S., Marcheggiani F., Cirilli I., Dludla P.V., Fiorini R., Pacetti D., Loizzo M.R., Lucci P. (2022). Bioavailability Study of Isothiocyanates and Other Bioactive Compounds of Brassica oleracea L. var. Italica Boiled or Steamed: Functional Food or Dietary Supplement?. Antioxidants.

[308] Rahman M.S. (2007). Allicin and Other Functional Active Components in Garlic: Health Benefits and Bioavailability. Int. J. Food Prop..

[309] Lawson L.D., Hunsaker S.M. (2018). Allicin Bioavailability and Bioequivalence from Garlic Supplements and Garlic Foods. Nutrients.

[310] Chhabria S., Desai K. (2018). Purification and characterisation of alliinase produced by Cupriavidus necator and its application for generation of cytotoxic agent: Allicin. Saudi J. Biol. Sci..

[311] Scalbert A., Williamson G. (2000). Dietary intake and bioavailability of polyphenols. J. Nutr..

[312] Padilla-González G.F., Grosskopf E., Sadgrove N.J., Simmonds M.S.J. (2022). Chemical Diversity of Flavan-3-Ols in Grape Seeds: Modulating Factors and Quality Requirements. Plants.

[313] Rudrapal M., Khairnar S.J., Khan J., Dukhyil A.B., Ansari M.A., Alomary M.N., Alshabrmi F.M., Palai S., Deb P.K., Devi R. (2022). Dietary polyphenols and their role in oxidative stress-induced human diseases: Insights into protective effects, antioxidant potentials and mechanism (s) of action. Front. Pharmacol..

[314] Nakamura Y., Miyoshi N. (2010). Electrophiles in foods: The current status of isothiocyanates and their chemical biology. Biosci. Biotechnol. Biochem..

[315] de Araújo F.F., de Paulo Farias D., Neri-Numa I.A., Pastore G.M. (2021). Polyphenols and their applications: An approach in food chemistry and innovation potential. Food Chem..

[316] Fraga C.G., Croft K.D., Kennedy D.O., Tomás-Barberán F.A. (2019). The effects of polyphenols and other bioactives on human health. Food Funct..

[317] Velderrain-Rodríguez G., Palafox-Carlos H., Wall-Medrano A., Ayala-Zavala J., Chen C.O., Robles-Sánchez M., Astiazaran-García H., Alvarez-Parrilla E., González-Aguilar G. (2014). Phenolic compounds: Their journey after intake. Food Funct..

[318] Teng H., Chen L. (2019). Polyphenols and bioavailability: An update. Crit. Rev. Food Sci. Nutr..

[319] Williamson G., Clifford M.N. (2017). Role of the small intestine, colon and microbiota in determining the metabolic fate of polyphenols. Biochem. Pharmacol..

[320] Chen H., Sang S. (2014). Biotransformation of tea polyphenols by gut microbiota. J. Funct. Foods.

[321] Aravind S.M., Wichienchot S., Tsao R., Ramakrishnan S., Chakkaravarthi S. (2021). Role of dietary polyphenols on gut microbiota, their metabolites and health benefits. Food Res. Int..

[322] Aura A.-M. (2008). Microbial metabolism of dietary phenolic compounds in the colon. Phytochem. Rev..

[323] Bermúdez-Soto M.-J., Tomás-Barberán F.-A., García-Conesa M.-T. (2007). Stability of polyphenols in chokeberry (*Aronia melanocarpa*) subjected to in vitro gastric and pancreatic digestion. Food Chem..

[324] Gabriel A., Chinenye O., Rudrapal M. (2023). Toxicity of Polyphenols Consumed as Food and Nutraceuticals: Remedies through Nanotherapeutic Approaches. Polyphenols: Food, Nutraceutical, and Nanotherapeutic Applications.

[325] Cianciosi D., Forbes-Hernández T.Y., Regolo L., Alvarez-Suarez J.M., Navarro-Hortal M.D., Xiao J., Quiles J.L., Battino M., Giampieri F. (2022). The reciprocal interaction between polyphenols and other dietary compounds: Impact on bioavailability, antioxidant capacity and other physico-chemical and nutritional parameters. Food Chem..

[326] Dima C., Assadpour E., Dima S., Jafari S.M. (2021). Nutraceutical nanodelivery; an insight into the bioaccessibility/bioavailability of different bioactive compounds loaded within nanocarriers. Crit. Rev. Food Sci. Nutr..

[327] D’Archivio M., Filesi C., Varì R., Scazzocchio B., Masella R. (2010). Bioavailability of the polyphenols: Status and controversies. Int. J. Mol. Sci..

[328] Domínguez-Avila J.A., Wall-Medrano A., Velderrain-Rodríguez G.R., Chen C.-Y.O., Salazar-López N.J., Robles-Sánchez M., González-Aguilar G.A. (2017). Gastrointestinal interactions, absorption, splanchnic metabolism and pharmacokinetics of orally ingested phenolic compounds. Food Funct..

[329] Day A.J., Cañada F.J., Díaz J.C., Kroon P.A., Mclauchlan R., Faulds C.B., Plumb G.W., Morgan M.R., Williamson G. (2000). Dietary flavonoid and isoflavone glycosides are hydrolysed by the lactase site of lactase phlorizin hydrolase. FEBS Lett..

[330] Chen L., Cao H., Xiao J. (2018). Polyphenols: Absorption, bioavailability, and metabolomics. Polyphenols: Properties, Recovery, and Applications.

[331] Majee C., Mazumder R., Choudhary A.N. (2023). An Insight into the Hepatoprotective Activity and Structure-activity Relationships of Flavonoids. Mini Rev. Med. Chem..

[332] Rodríguez-Daza M.C., Pulido-Mateos E.C., Lupien-Meilleur J., Guyonnet D., Desjardins Y., Roy D. (2021). Polyphenol-mediated gut microbiota modulation: Toward prebiotics and further. Front. Nutr..

[333] Sharma R., Diwan B., Singh B.P., Kulshrestha S. (2022). Probiotic fermentation of polyphenols: Potential sources of novel functional foods. Food Prod. Process. Nutr..

[334] Rana A., Samtiya M., Dhewa T., Mishra V., Aluko R.E. (2022). Health benefits of polyphenols: A concise review. J. Food Biochem..

